# Supplementary figures and images for: Non-linear archetypal analysis of single-cell RNA-seq data by deep autoencoders
Source: PLoS Comput Biol. 2022 Apr 1;18(4):e1010025. doi: 10.1371/journal.pcbi.1010025 (PMC9007392; doi:10.1371/journal.pcbi.1010025)

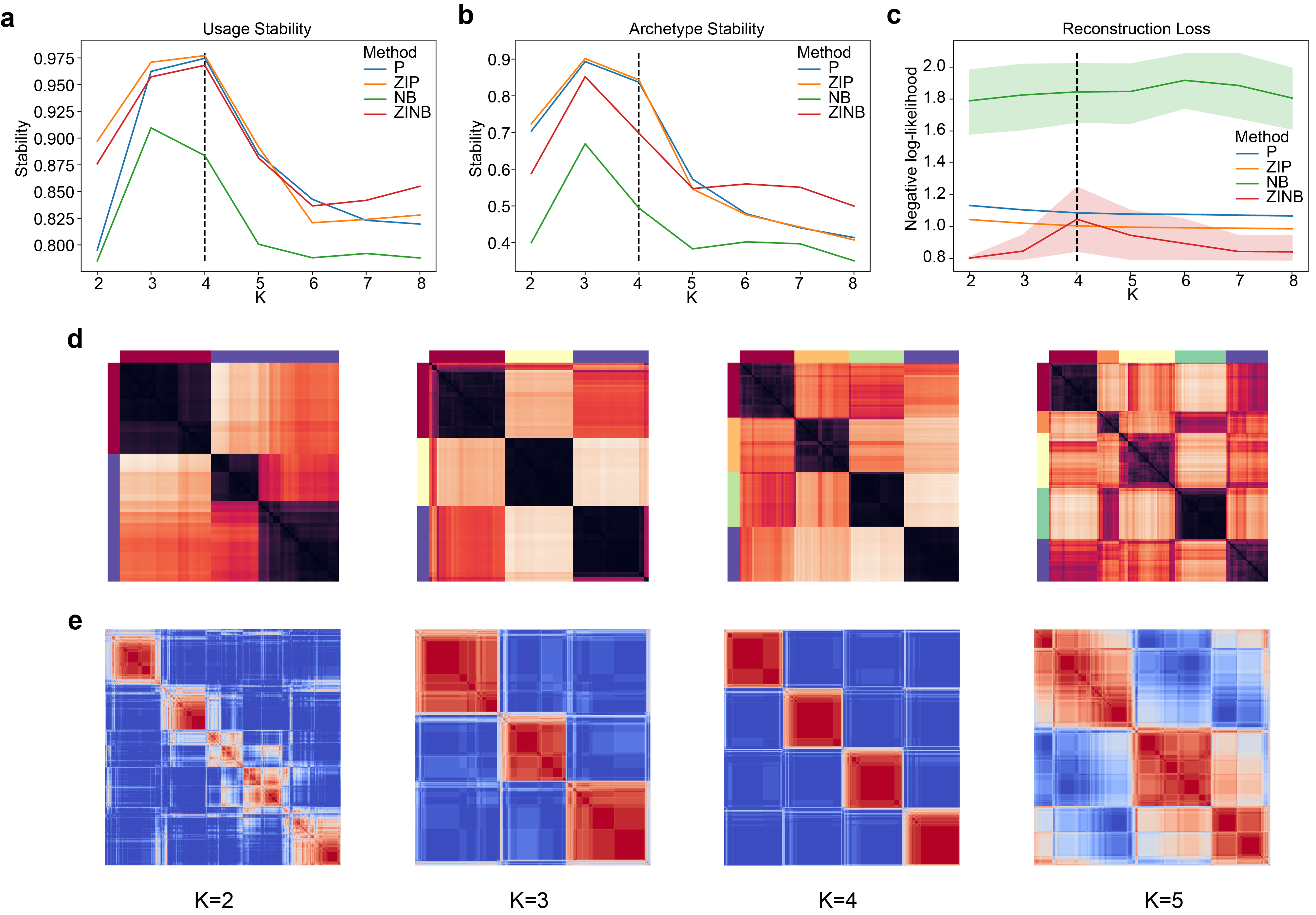

Supplement: S1 Fig — (a) Stability of usage, (b) stability of archetypes and (c) reconstruction loss across different Ks for four count distributions. (d) Distance matrix showing archetype stability under K = 2–5. The darker the color the smaller the distance. (e) Consensus clustering matrices showing the usage stability under K = 2–5. The warmer the color the large the similarity. (TIF) [file pcbi.1010025.s001.tif]

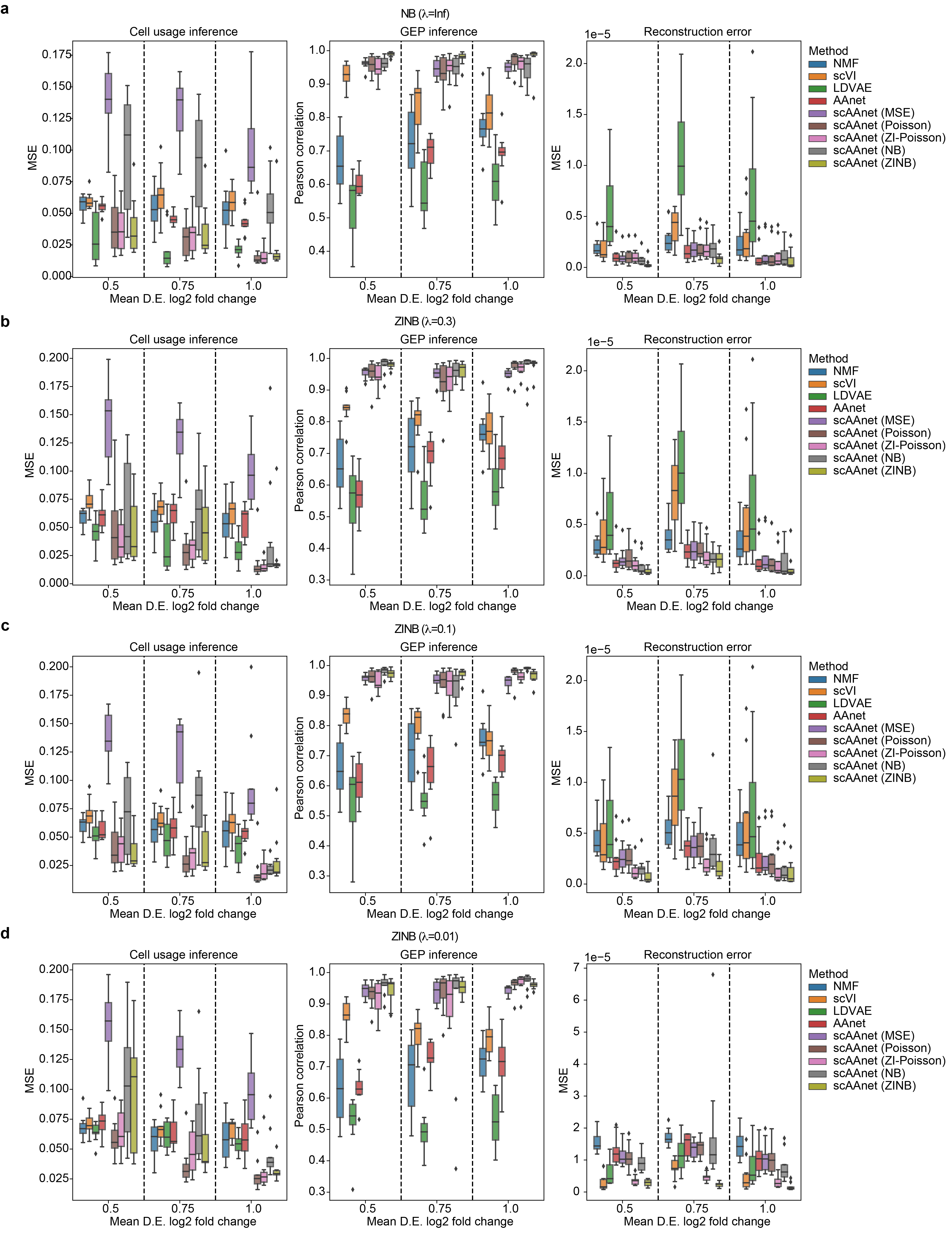

Supplement: S2 Fig — (a) Results from datasets that were simulated under NB distributions. (b) Results under ZINB distributions with λ = 0.3. (c) Results under ZINB distributions with λ = 0.1. (d) Results under ZINB distributions with λ = 0.01. For each panel, figures from left to right are MSE between inferred cell usages and true usages of the 4 GEPs, Pearson correlation between inferred GEPs and true GEPs, and MSE between reconstructed scaled means and true means. Each box and whisker plot was plotted based on ten simulated datasets. Central lines represent medians, boxes represent the IQR, and the upper/lower whisker represents the largest/smallest value no further than 1.5 × IQR. D.E.: differential expression. (TIF) [file pcbi.1010025.s002.tif]

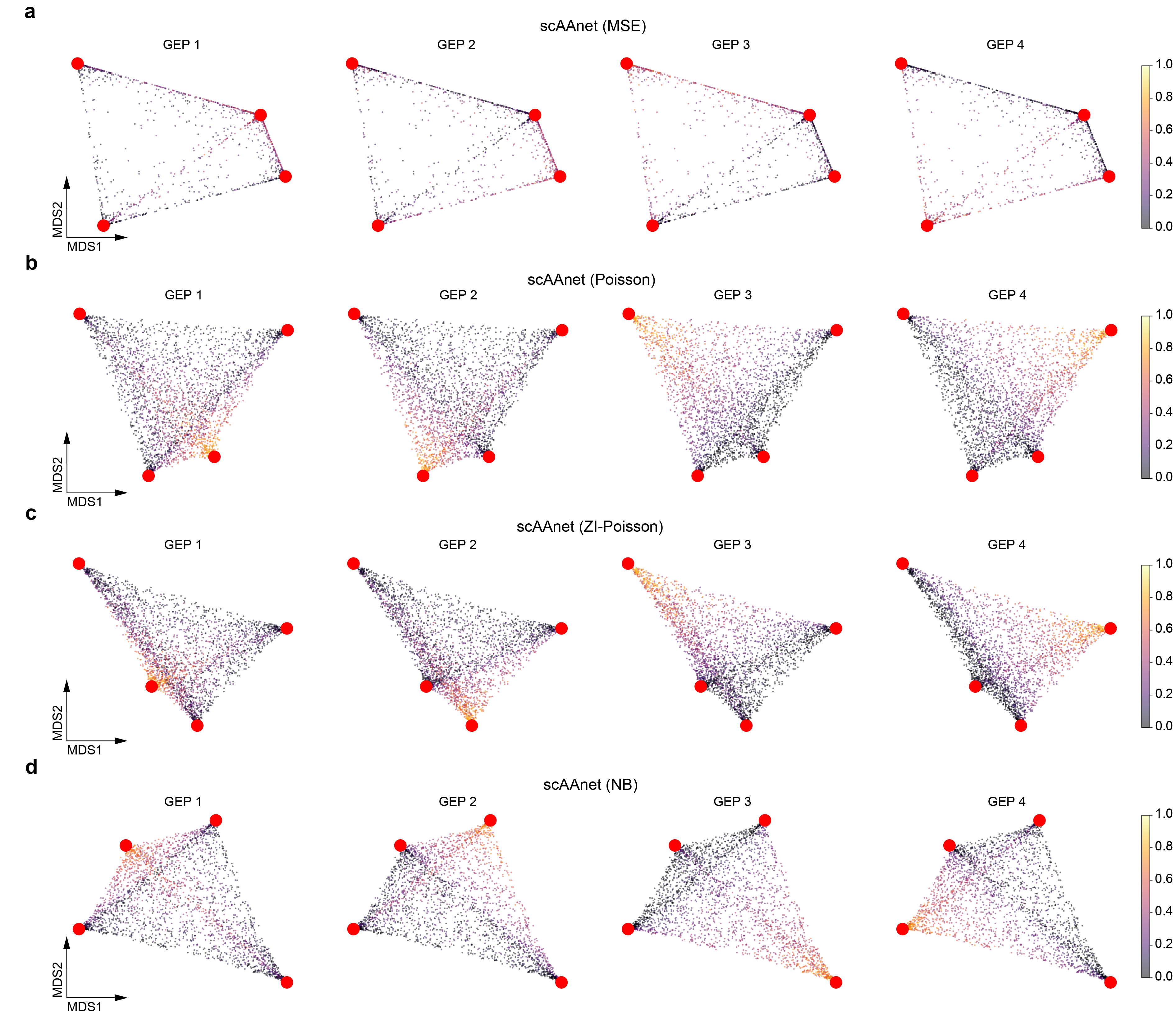

Supplement: S3 Fig — (a) scAAnet with MSE, (b) scAAnet with Poisson, (c) scAAnet with ZIP, and (d) scAAnet with NB distributions as the reconstruction error term. Red dots are the locations of archetypes. Figures in each column are colored by the true cell usage of the corresponding archetype (GEP). (TIF) [file pcbi.1010025.s003.tif]

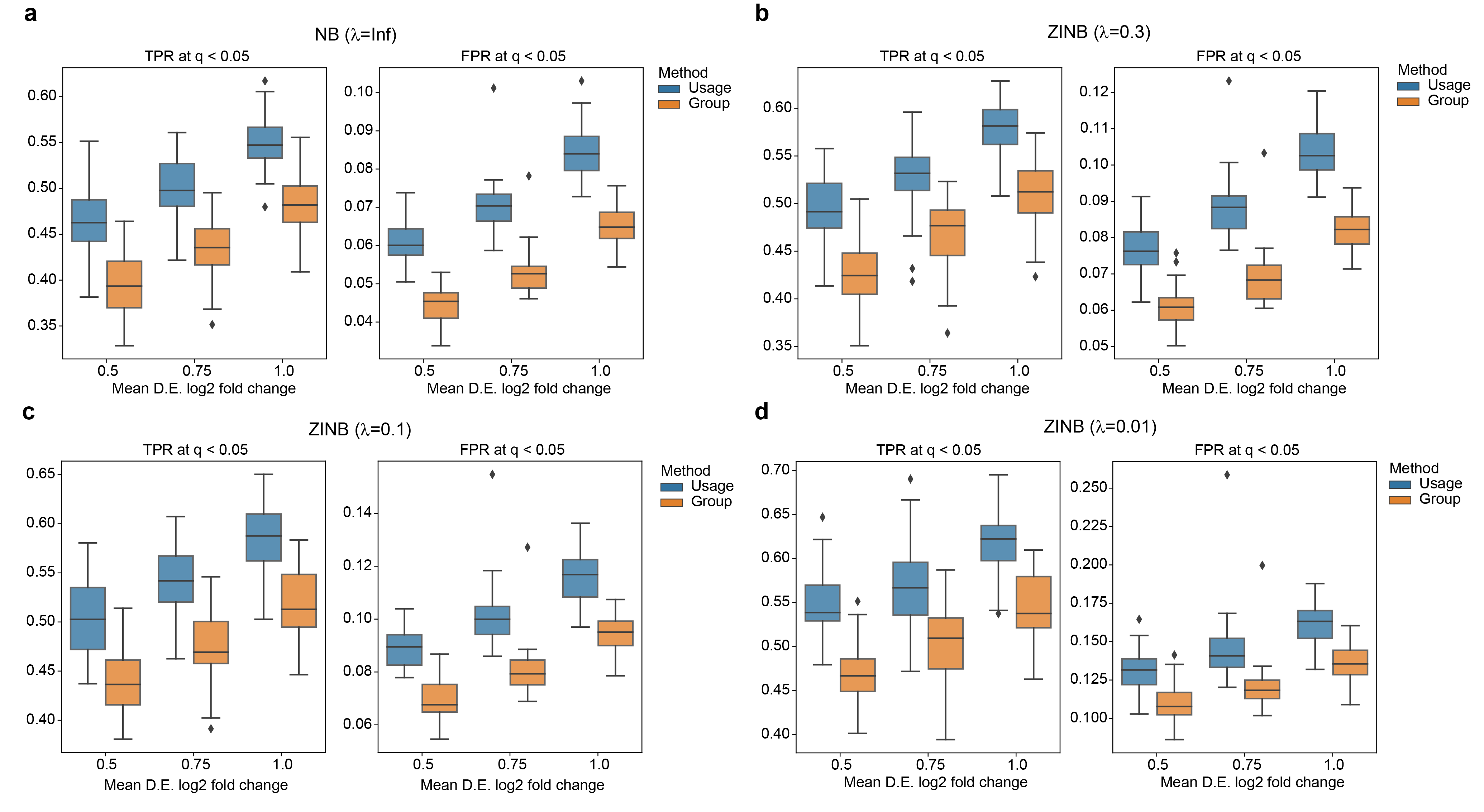

Supplement: S4 Fig — (a) Datasets were simulated under NB, (b) ZINB with λ = 0.3, (c) ZINB with λ = 0.1, and (d) ZINB with λ = 0.01. For each panel, figures from left to right are TPR at q-value < 0.05 and FPR at q-value < 0.05 calculated across different signal-to-noise ratio levels. Bonferroni correction was used to obtain q-values. Each box and whisker plot was plotted based on ten simulated datasets. Central lines represent medians, boxes represent the IQR, and the upper/lower whisker represents the largest/smallest value no further than 1.5 × IQR. TPR: true positive rate; FPR: false positive rate. (TIF) [file pcbi.1010025.s004.tif]

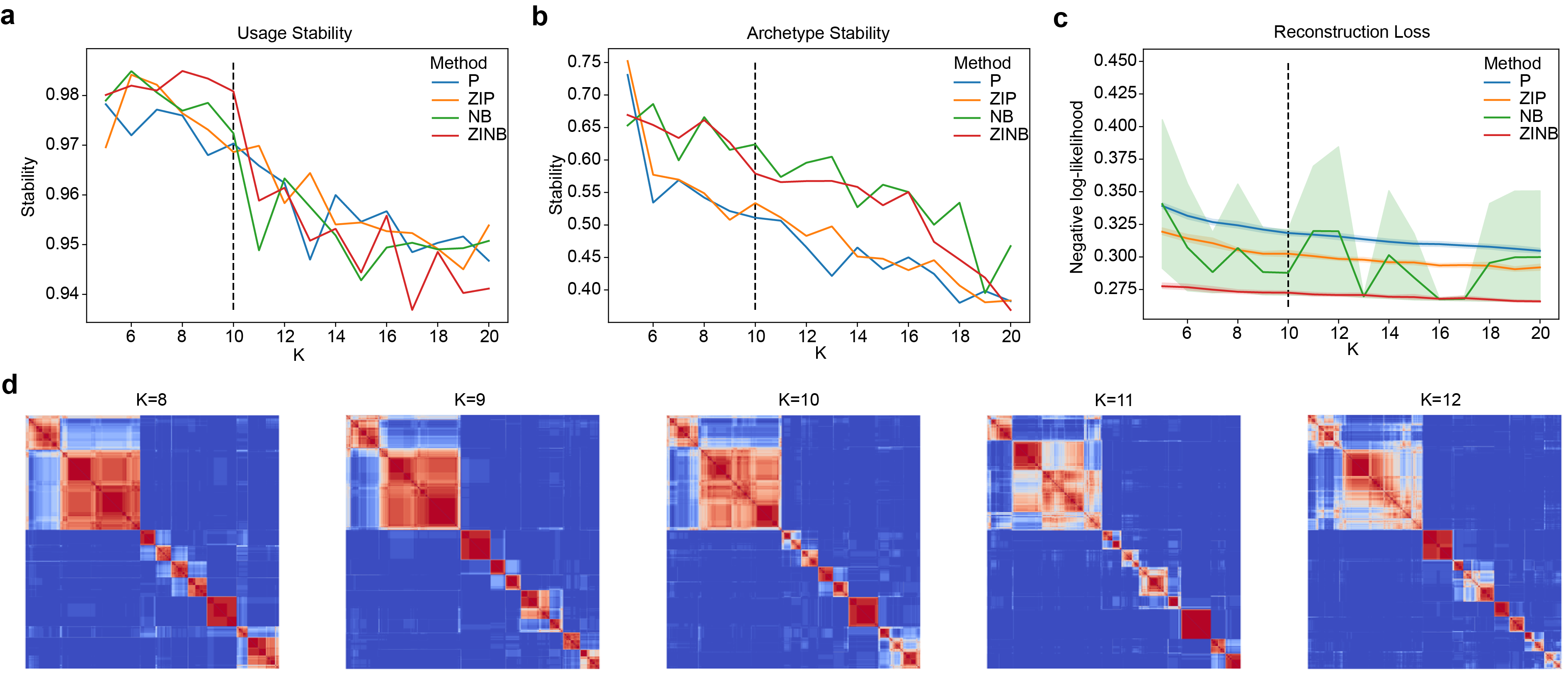

Supplement: S5 Fig — (a) Stability of usage, (b) stability of archetypes and (c) reconstruction loss across different Ks for four count distributions. (d) Consensus clustering matrices showing the usage stability under K = 8–12. The warmer the color the large the similarity. (TIF) [file pcbi.1010025.s005.tif]

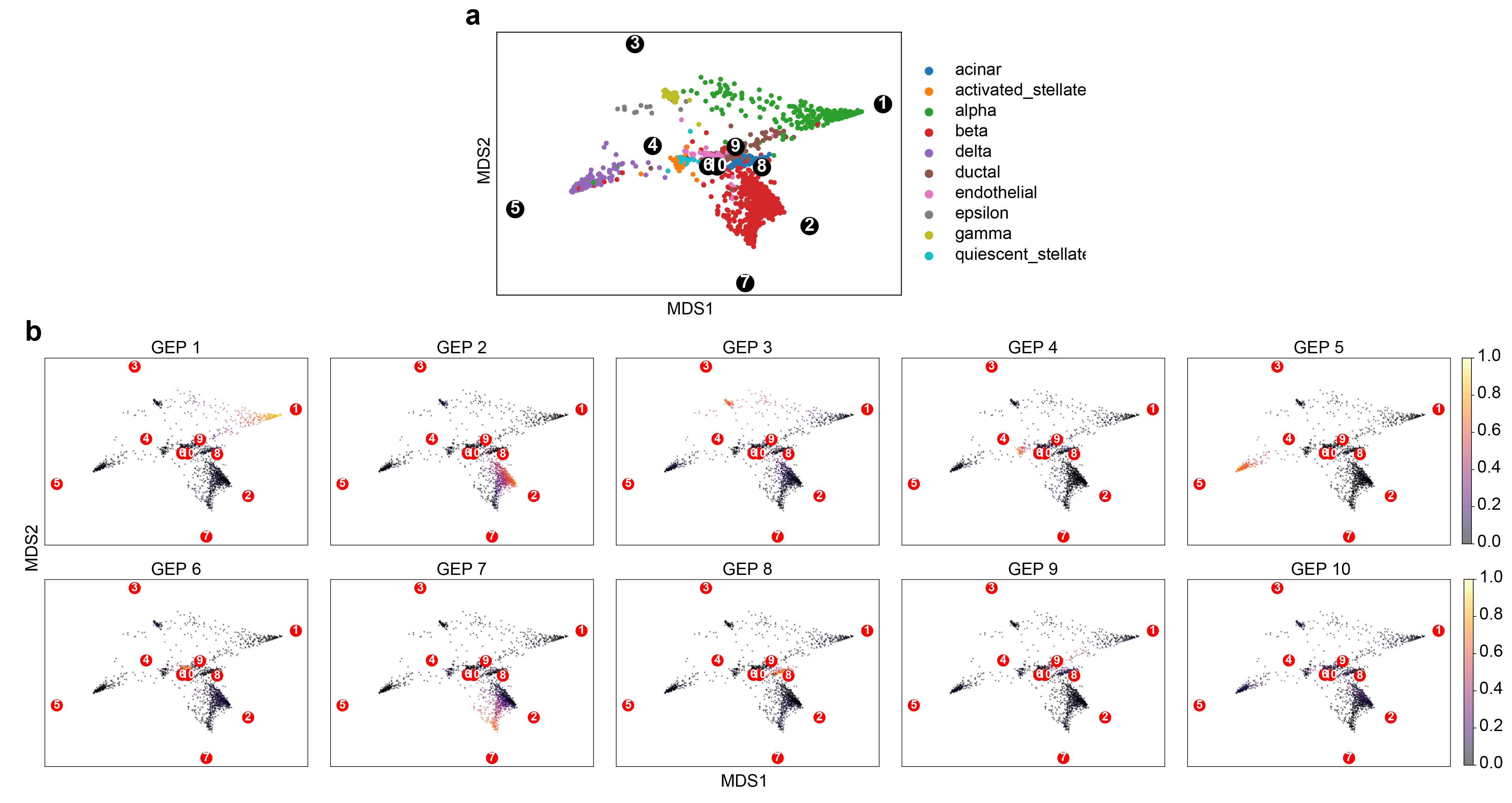

Supplement: S6 Fig — (a) MDS visualization colored by cell types. Locations of inferred archetypes are shown as black dots. (b) MDS visualization colored by the inferred usage of the ten GEPs. Locations of inferred archetypes are shown as red dots. (TIF) [file pcbi.1010025.s006.tif]

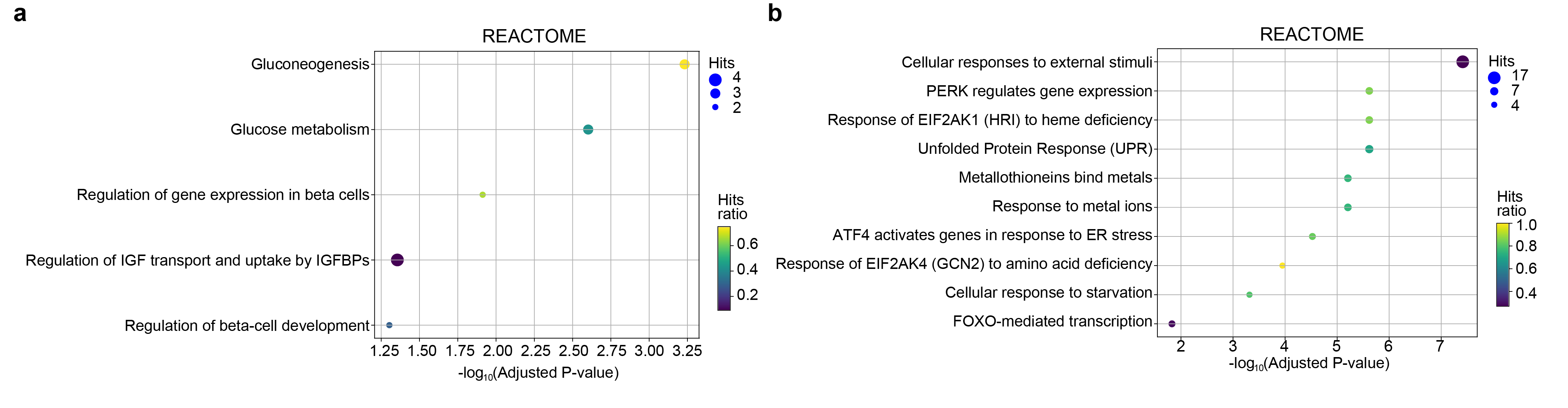

Supplement: S7 Fig — (a) Top enriched terms identified using significantly upregulated genes in GEP 2. (b) Top 10 enriched terms identified using significantly upregulated genes in GEP 7. (TIF) [file pcbi.1010025.s007.tif]

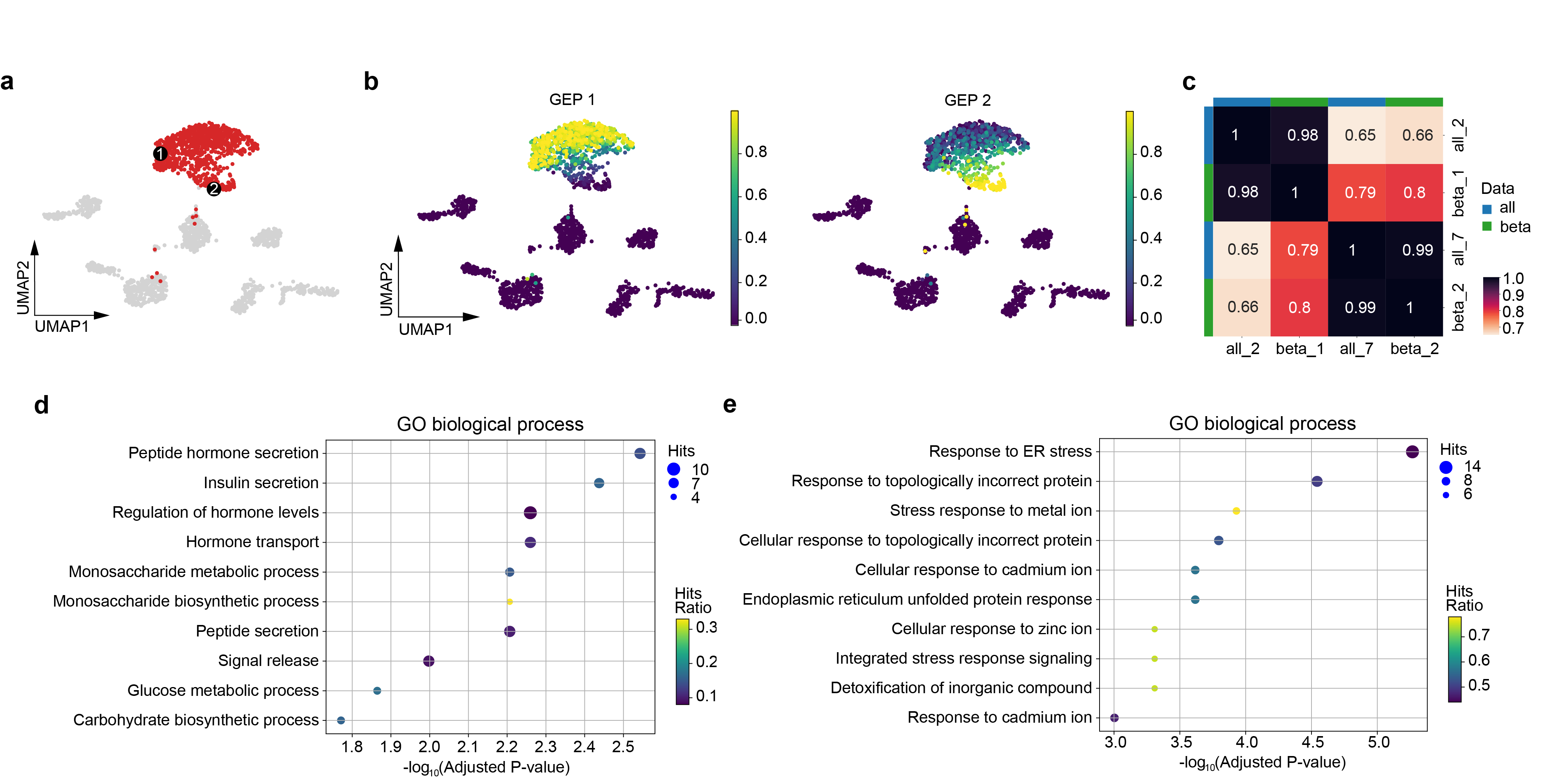

Supplement: S8 Fig — (a) The UMAP visualization of the pancreatic islet scRNA-seq dataset, with beta cells highlighted in red. Black dots are locations of cells that have the largest usage of the 2 GEPs (marked in Arabic numerals). (b) UMAPs colored by inferred cell usage for each GEP. (c) Heatmap showing the Pearson correlations among GEP 1 and GEP 2 identified using beta cells only and GEP 2 and GEP 7 identified using the entire dataset. (d) The top 10 enriched GO biological process terms using 35 significantly upregulated genes of GEP 1. (e) The top 10 enriched GO biological process terms using 130 significantly upregulated genes of GEP 2. (TIF) [file pcbi.1010025.s008.tif]

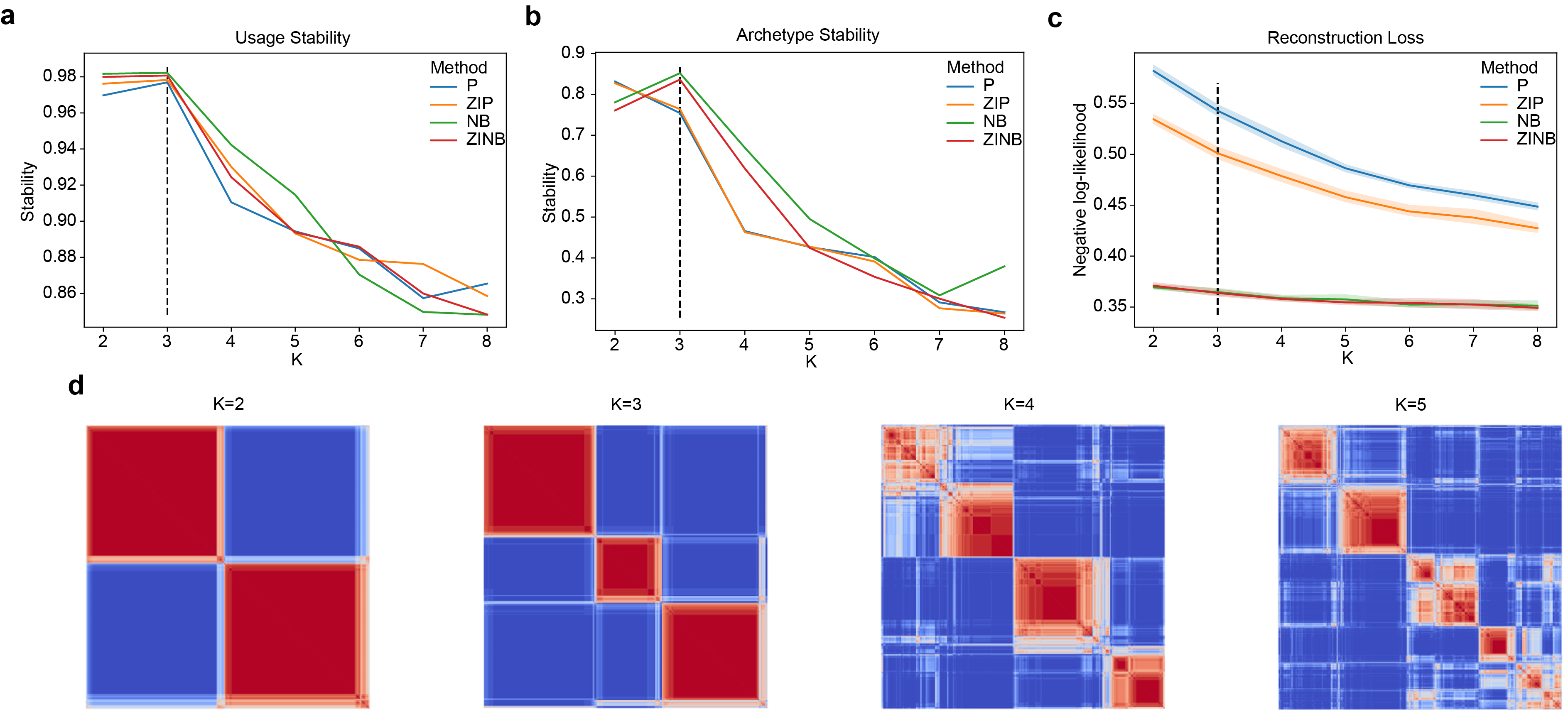

Supplement: S9 Fig — (a) Stability of usage, (b) stability of archetypes and (c) reconstruction loss across different Ks for four count distributions. (d) Consensus clustering matrices showing the usage stability under K = 2–5. The warmer the color the large the similarity. (TIF) [file pcbi.1010025.s009.tif]

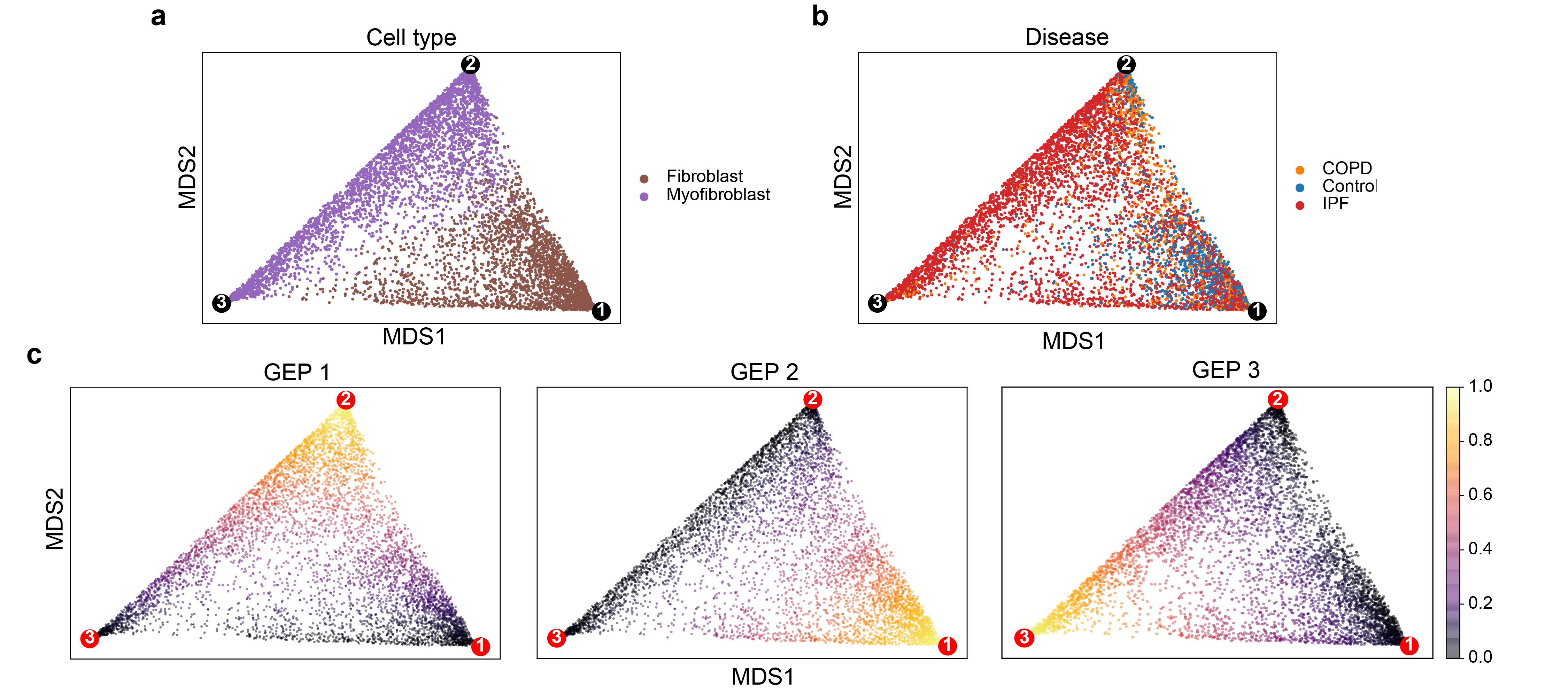

Supplement: S10 Fig — (a) MDS visualization colored by cell types. (b) MDS visualization colored by disease groups. Locations of inferred archetypes in a and b are shown as black dots. (c) MDS visualization colored by the inferred usage of the three GEPs. Locations of inferred archetypes are shown as red dots. (TIF) [file pcbi.1010025.s010.tif]

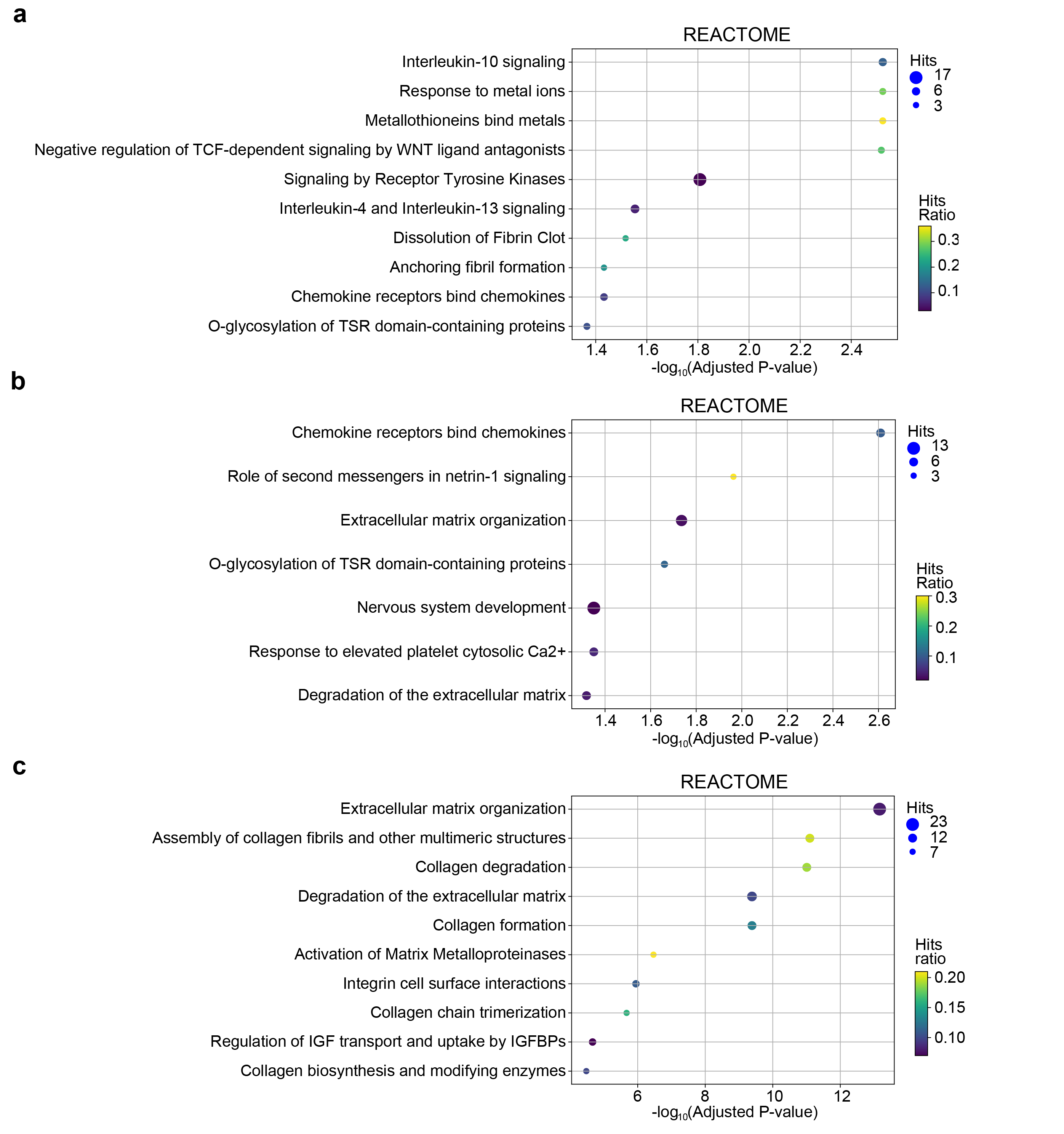

Supplement: S11 Fig — (a) Top enriched terms significantly upregulated genes in GEP 1, (b) GEP 2 and (c) GEP 3, respectively. (TIF) [file pcbi.1010025.s011.tif]

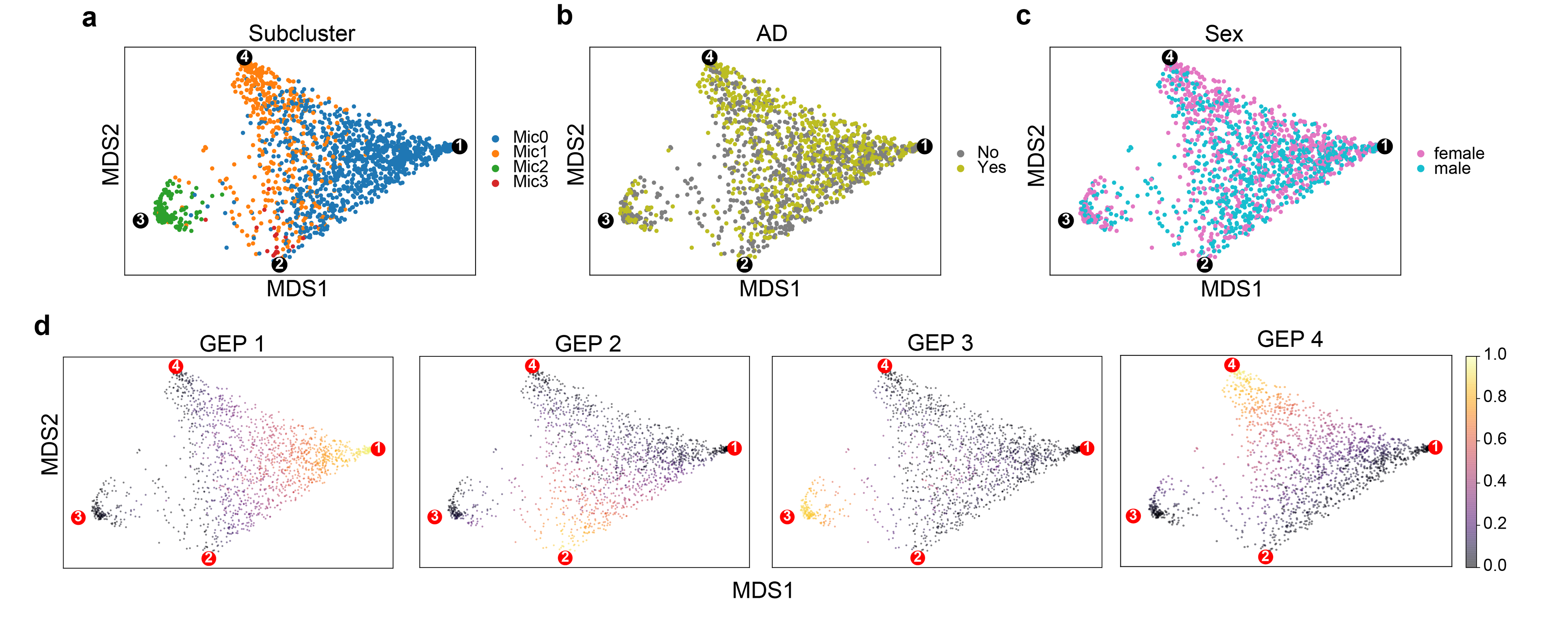

Supplement: S12 Fig — (a) MDS visualization colored by subclusters. (b) MDS visualization colored by AD pathology groups. (c) MDS visualization colored by sexes. Locations of inferred archetypes in a, b and c are shown as black dots. (d) MDS visualization colored by the inferred usage of the four GEPs. Locations of inferred archetypes are shown as red dots. (TIF) [file pcbi.1010025.s012.tif]

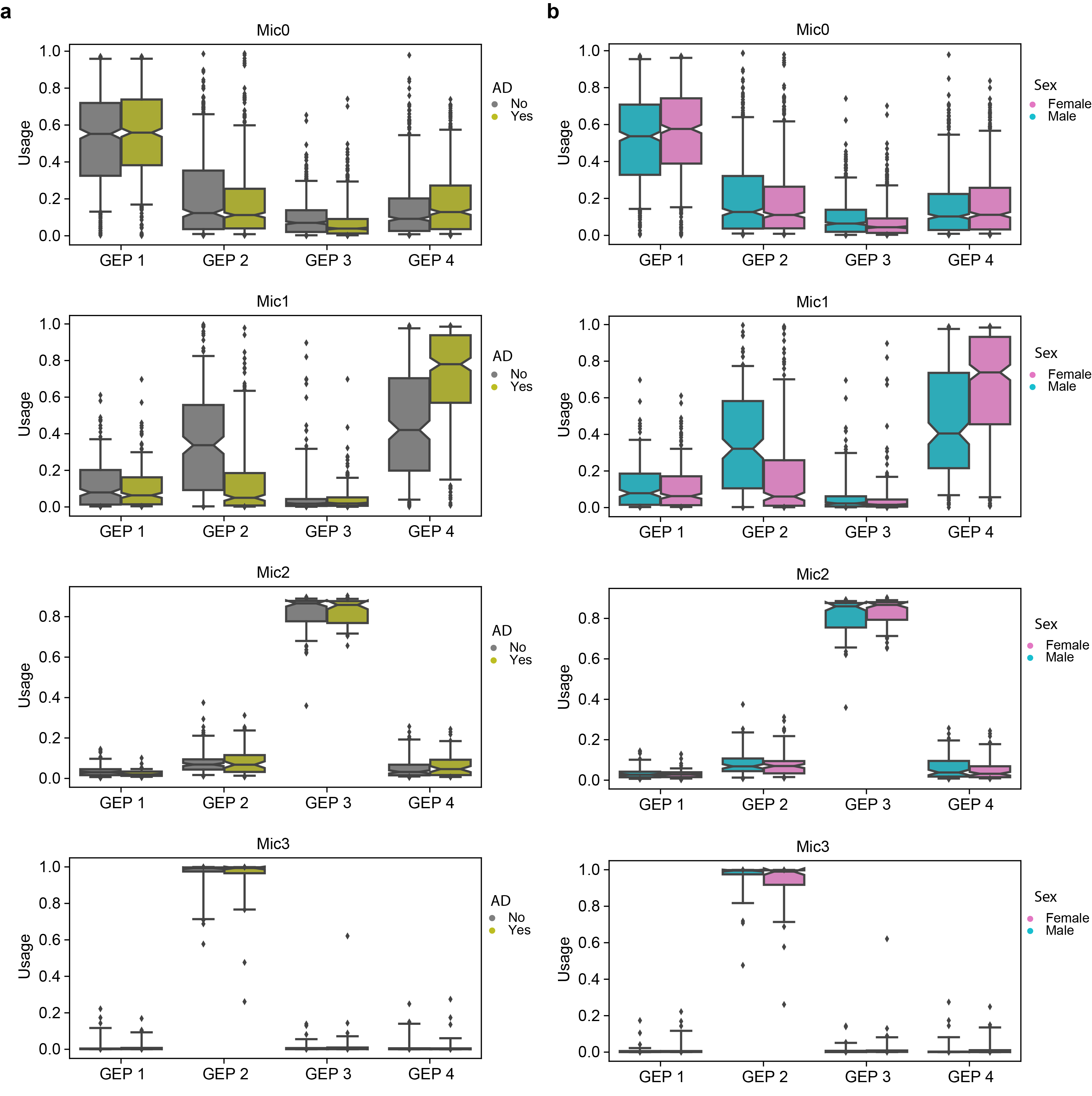

Supplement: S13 Fig — Box and whisker plot of the usage of each GEP in cells of subcluster Mic0, Mic1, Mic2, and Mic3 (from top to bottom), colored by (a) AD pathology group and by (b) sex, respectively. Central lines represent medians, boxes represent the IQR, and whiskers represent the 5th and 95th quantiles. (TIF) [file pcbi.1010025.s013.tif]

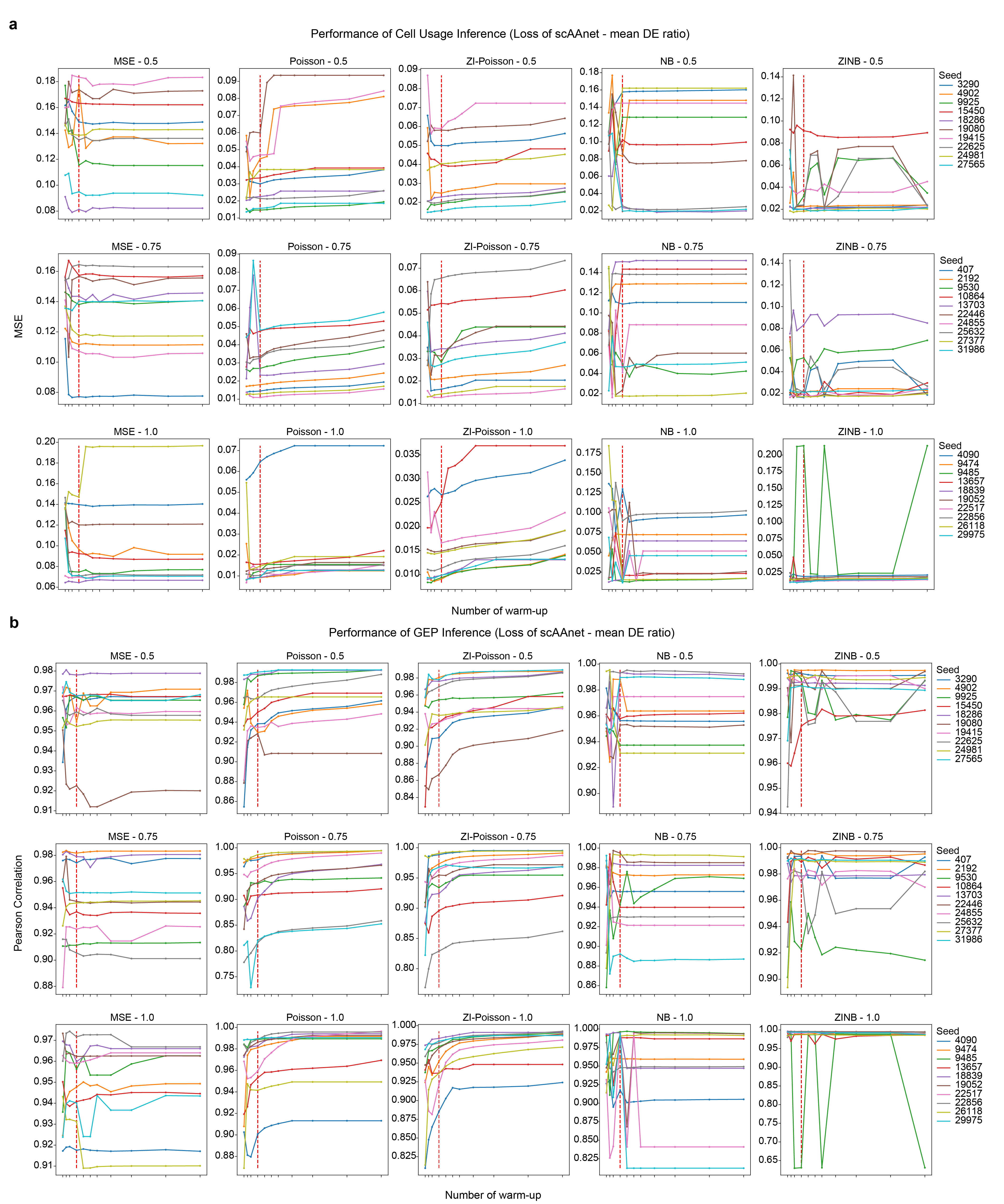

Supplement: S14 Fig — (a) Cell usage inference performance. MSE between inferred usage and true usage was calculated. (b) GEP inference performance. Pearson correlations between inferred GEPs and true GEPs were calculated. For both a and b, red dashed lines are vertical lines plotted at the number of warm-up periods being 20. Ticks along x-axis are numbers of warm-up periods (0, 5, 10, 20, 30, 40, 50, 70, 100, 150, and 200). The total number of epochs was fixed at 200 in this experiment, so if the number of warm-up period is 200, it means Zfix is not allowed to be trainable. (TIF) [file pcbi.1010025.s014.tif]

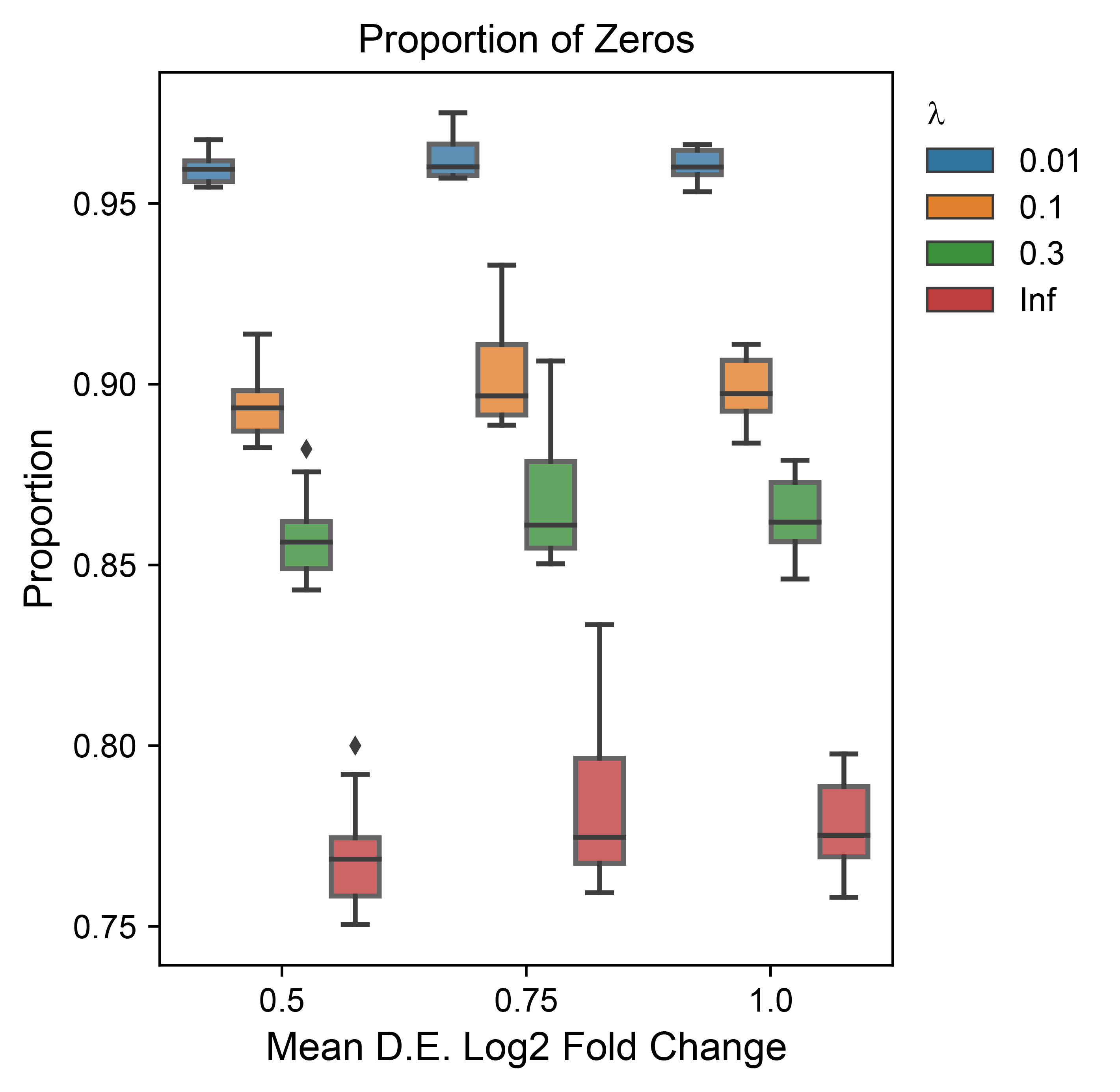

Supplement: S15 Fig — Each box and whisker plot was plotted based on ten simulated datasets. Central lines represent medians, boxes represent the interquartile range (IQR), and the upper/lower whisker represents the largest/smallest value no further than 1.5 × IQR. D.E.: differential expression. (TIF) [file pcbi.1010025.s015.tif]

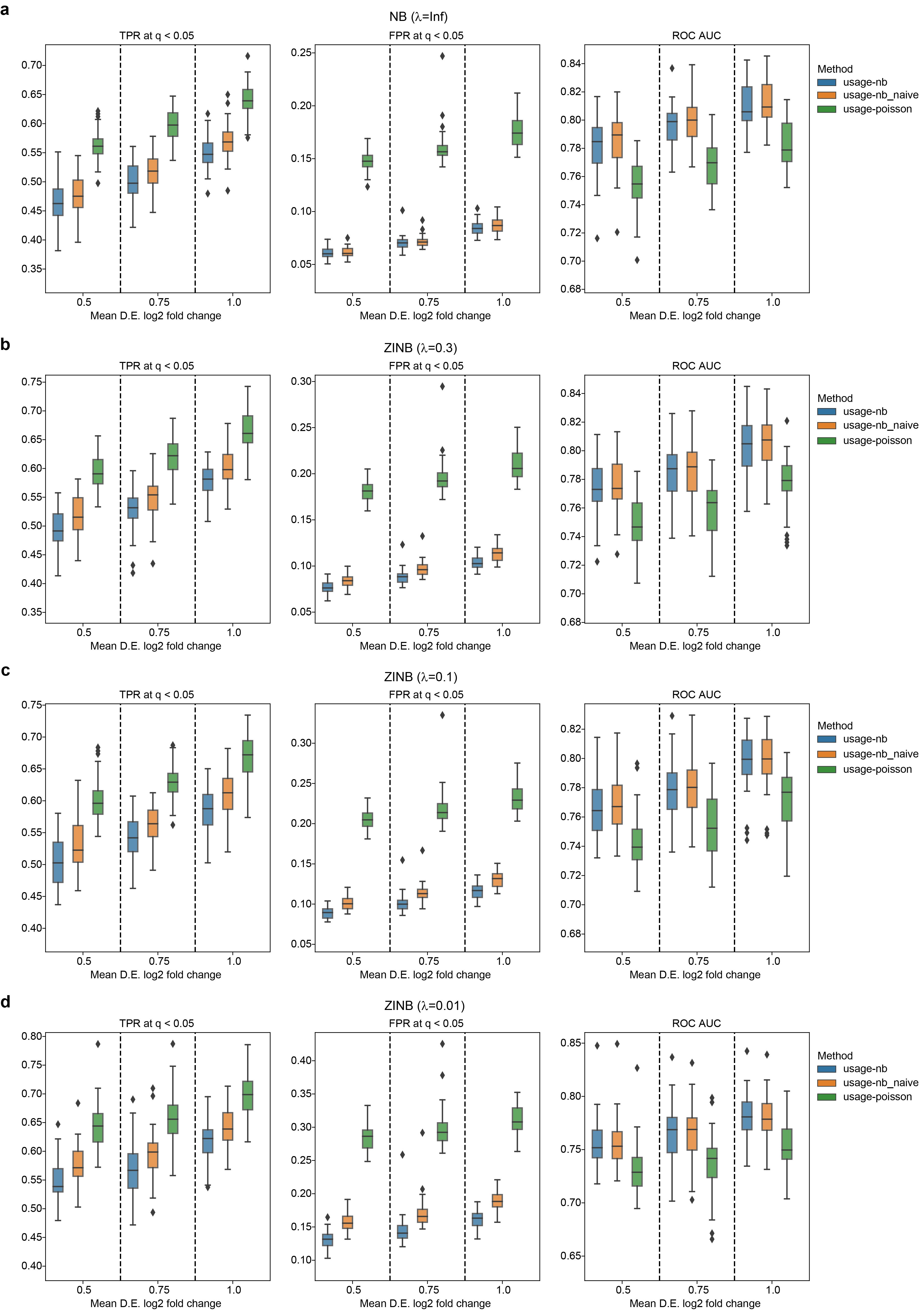

Supplement: S16 Fig — (a) Datasets were simulated under NB, (b) ZINB with λ = 0.3, (c) ZINB with λ = 0.1, and (d) ZINB with λ = 0.01. For each panel, figures from left to right are TPR at q-value < 0.05 and FPR at q-value < 0.05 calculated across different signal-to-noise ratio levels. Bonferroni correction was used to obtain q-values. Each box and whisker plot was plotted based on ten simulated datasets. Central lines represent medians, boxes represent the IQR, and the upper/lower whisker represents the largest/smallest value no further than 1.5 × IQR. TPR: true positive rate; FPR: false positive rate; AUC: area under curve. (TIF) [file pcbi.1010025.s016.tif]

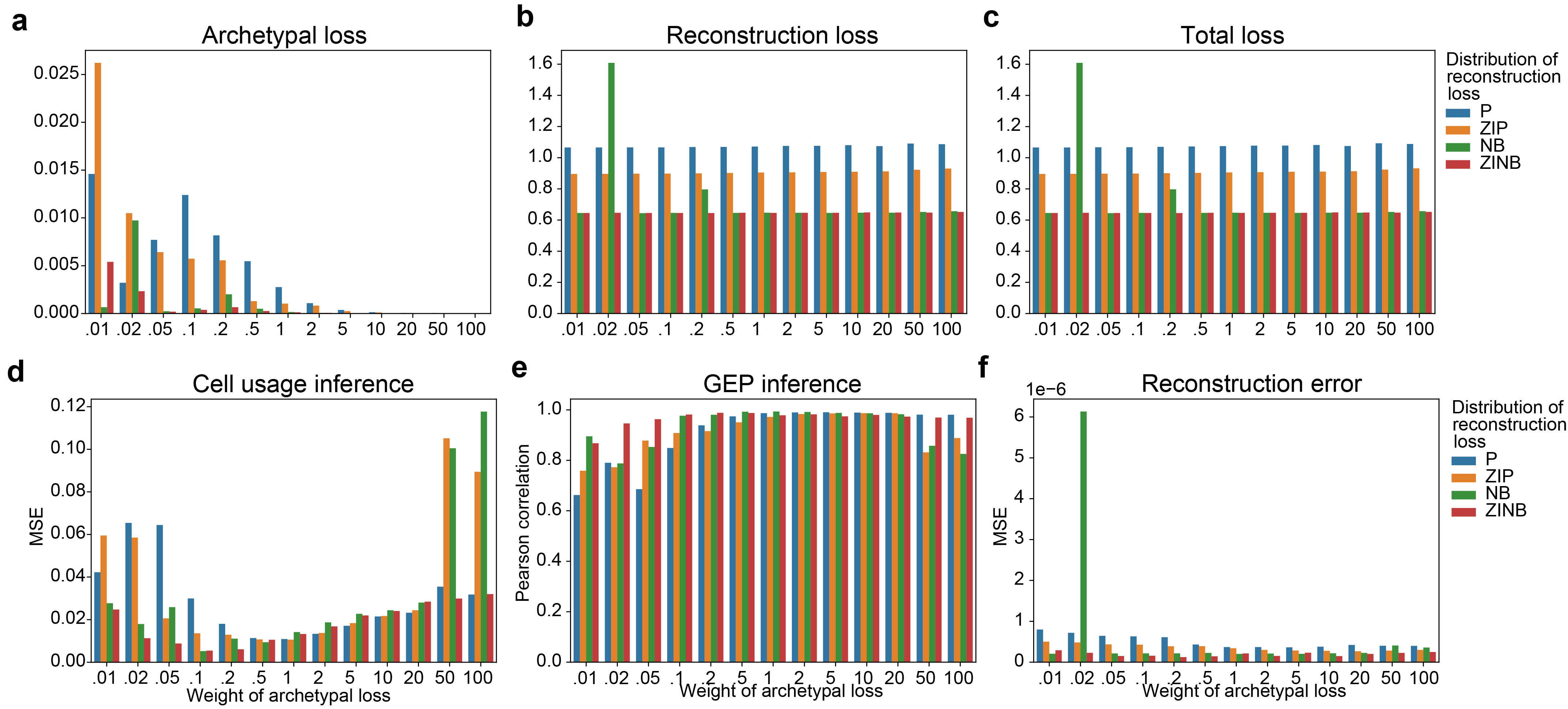

Supplement: S17 Fig — (a) Change of the archetypal loss. (b) Change of the reconstruction loss. (c) Change of the total loss. (d) Performance on cell usage inference across different weights. (e) Performance on GEP inference across different weights. (f) Performance on reconstruction accuracy across different weights. (TIF) [file pcbi.1010025.s017.tif]

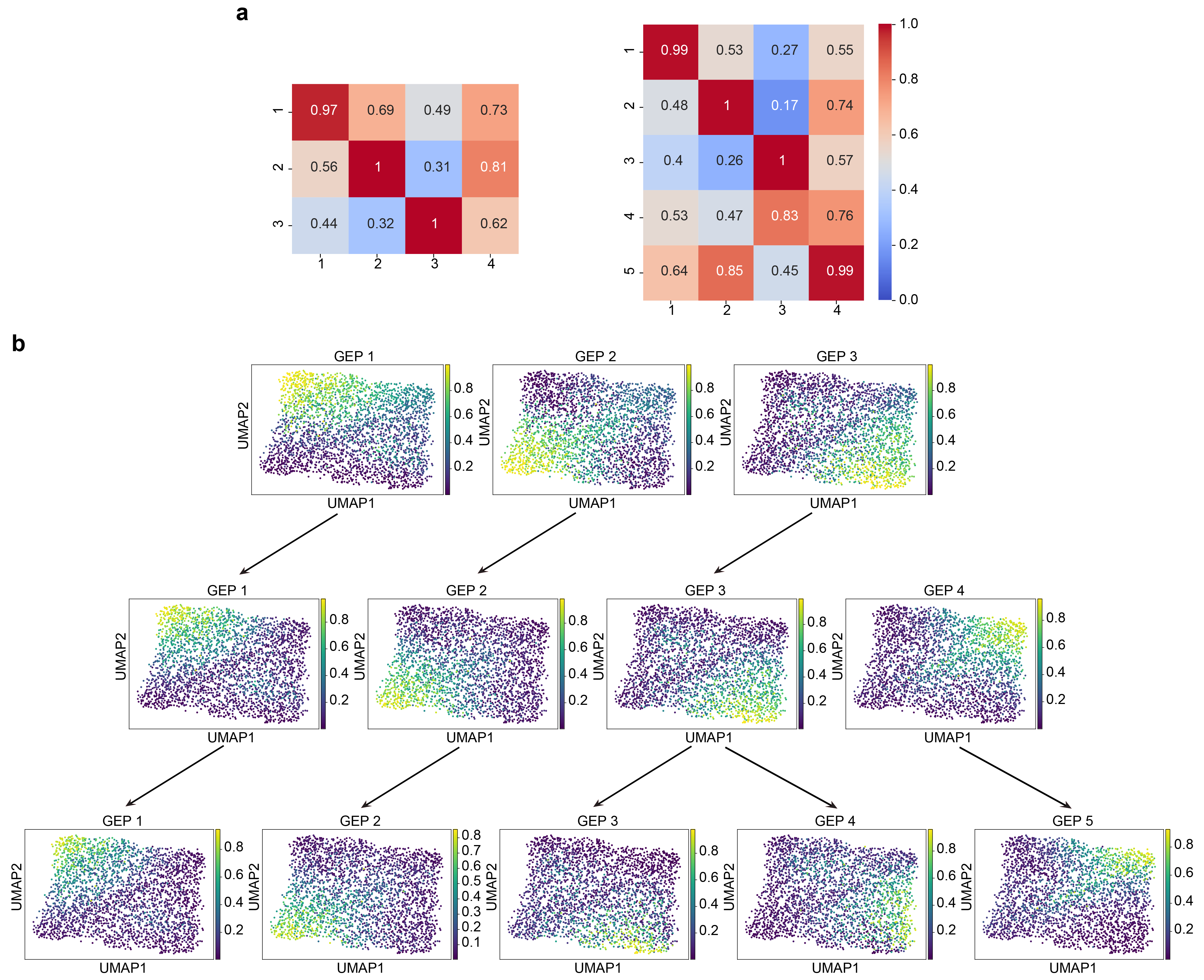

Supplement: S18 Fig — (a) Correlation heatmap between pairs of archetypes inferred using K = 3 and K = 4 (left), and between inferred using K = 5 and K = 4 (right). (b) UMAPs colored by the inferred GEP usage. From top to bottom are results based on K being 3, 4, and 5, respectively. Orange arrows indicate the progression of GEPs as K increases. (TIF) [file pcbi.1010025.s018.tif]

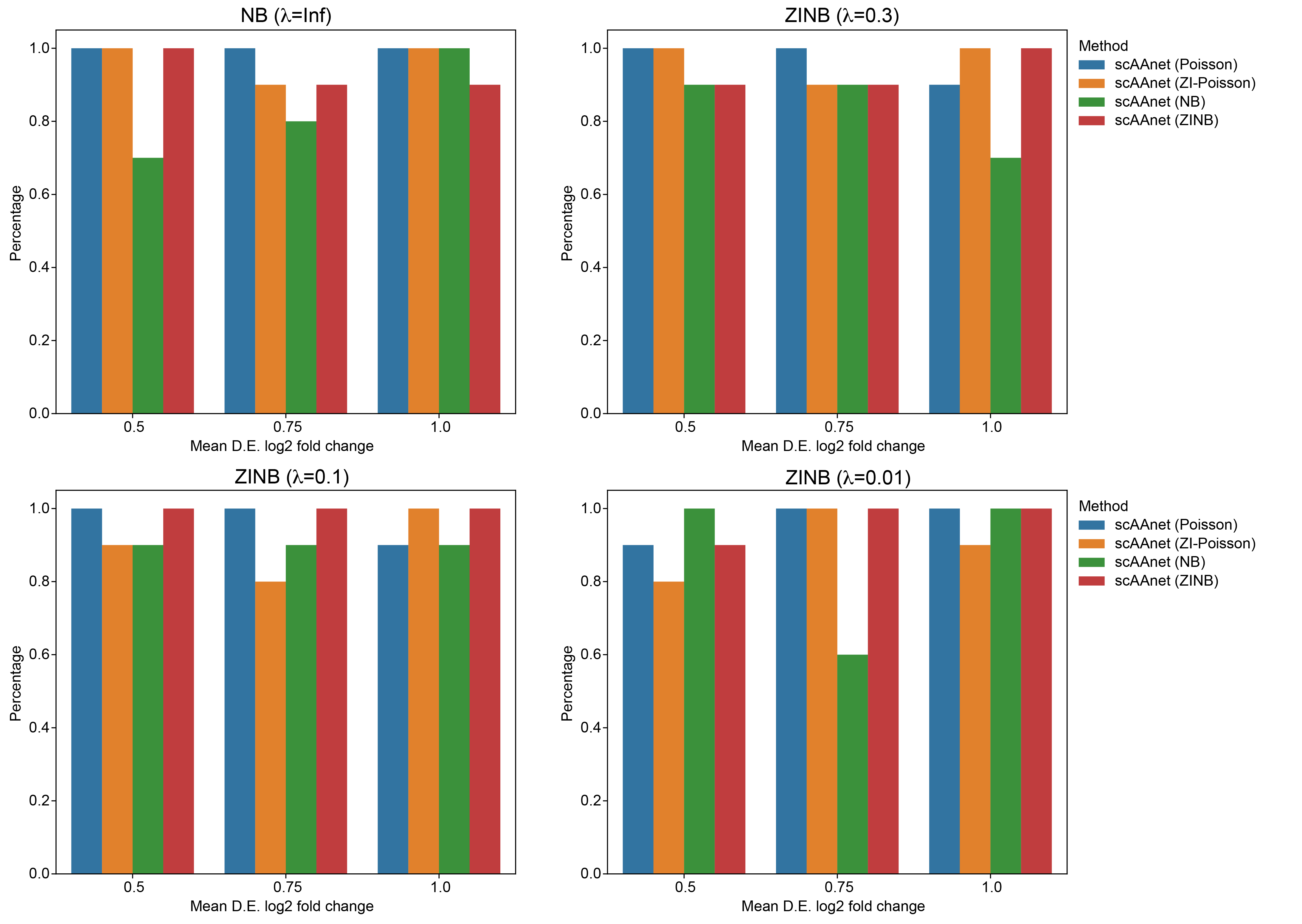

Supplement: S19 Fig — Each bar represents a simulation setting where ten repeated experiments were run under different seeds. The percentage on the y-axis was calculated as the number of experiments where GEPs inferred under K = 3 constituted a perfect subset of GEPs inferred under K = 4 divided by ten. A perfect subset was defined as no overlaps among the GEPs inferred under K = 4 that had the largest correlations with the GEPs inferred under K = 3. (TIF) [file pcbi.1010025.s019.tif]

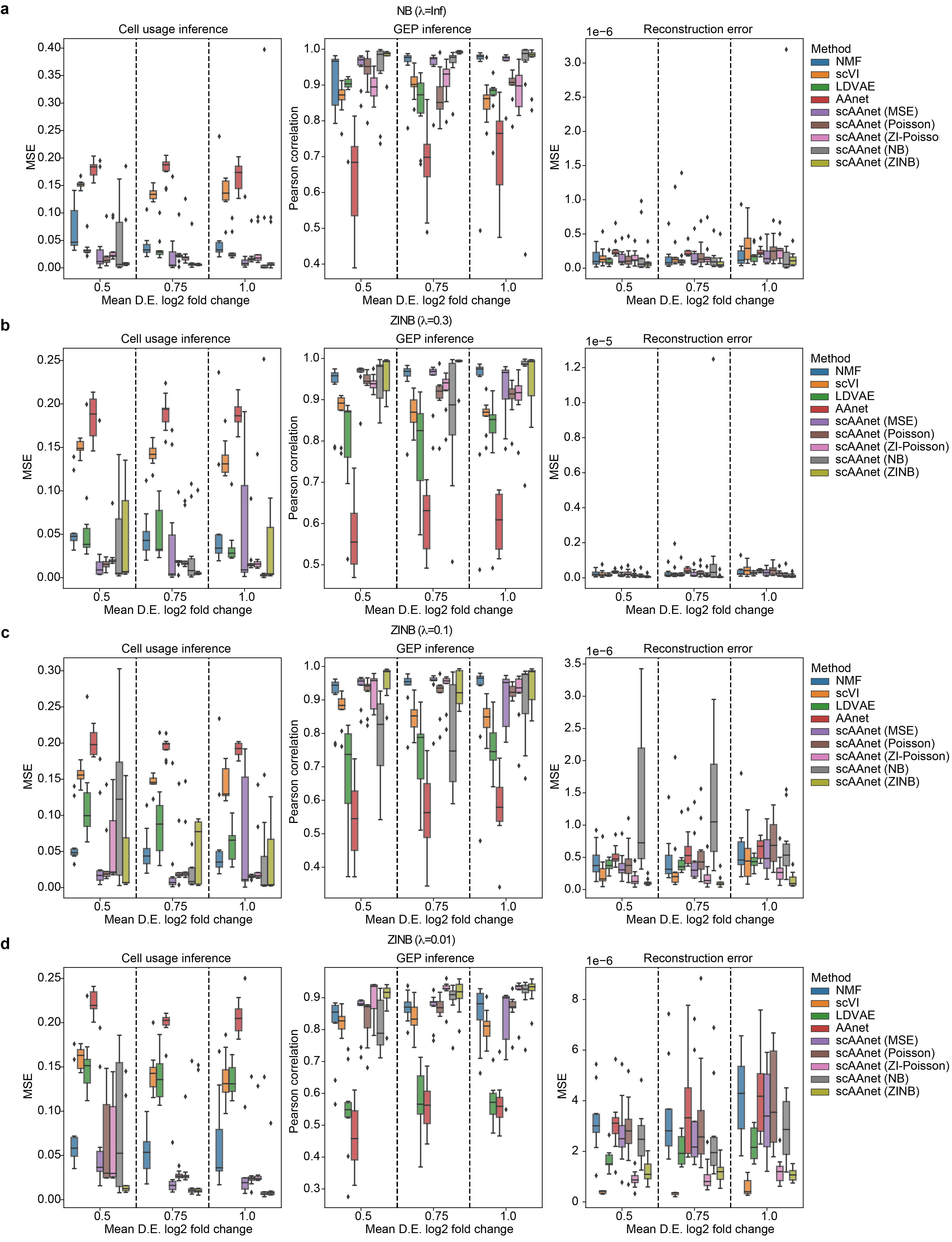

Supplement: S20 Fig — (a) Results from datasets that were simulated under NB distributions. (b) Results under ZINB distributions with λ = 0.3. (c) Results under ZINB distributions with λ = 0.1. (d) Results under ZINB distributions with λ = 0.01. Other details are the same as S2 Fig. (TIF) [file pcbi.1010025.s020.tif]

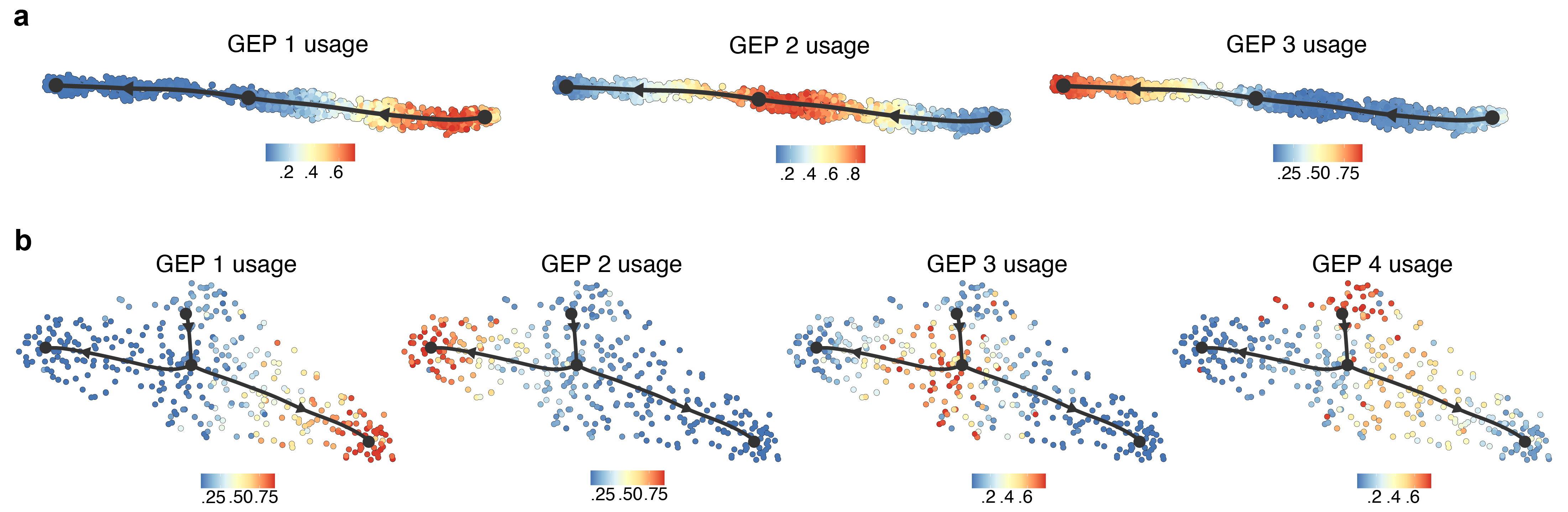

Supplement: S21 Fig — (a) Linear trajectory with three milestones colored by inferred GEP usage. (b) Bifurcation trajectory with four milestones colored by inferred GEP usage. Milestones are marked as black dots and backbone of the trajectory is shown in black curves. (TIF) [file pcbi.1010025.s021.tif]

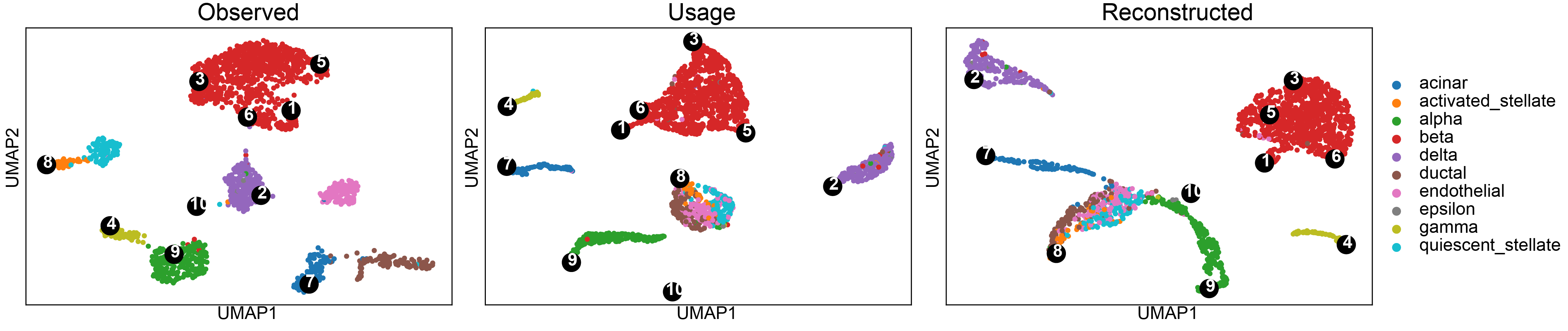

Supplement: S22 Fig — From left to right are UMAPs plotted using the top 35 PCs of the observed scRNA-seq data, using the inferred cell usage matrix, and using the reconstructed expression matrix. UMAPs are colored by 10 known cell clusters. Black dots are locations of cells that have the largest usage of the corresponding GEP (marked in Arabic numerals). (TIF) [file pcbi.1010025.s022.tif]

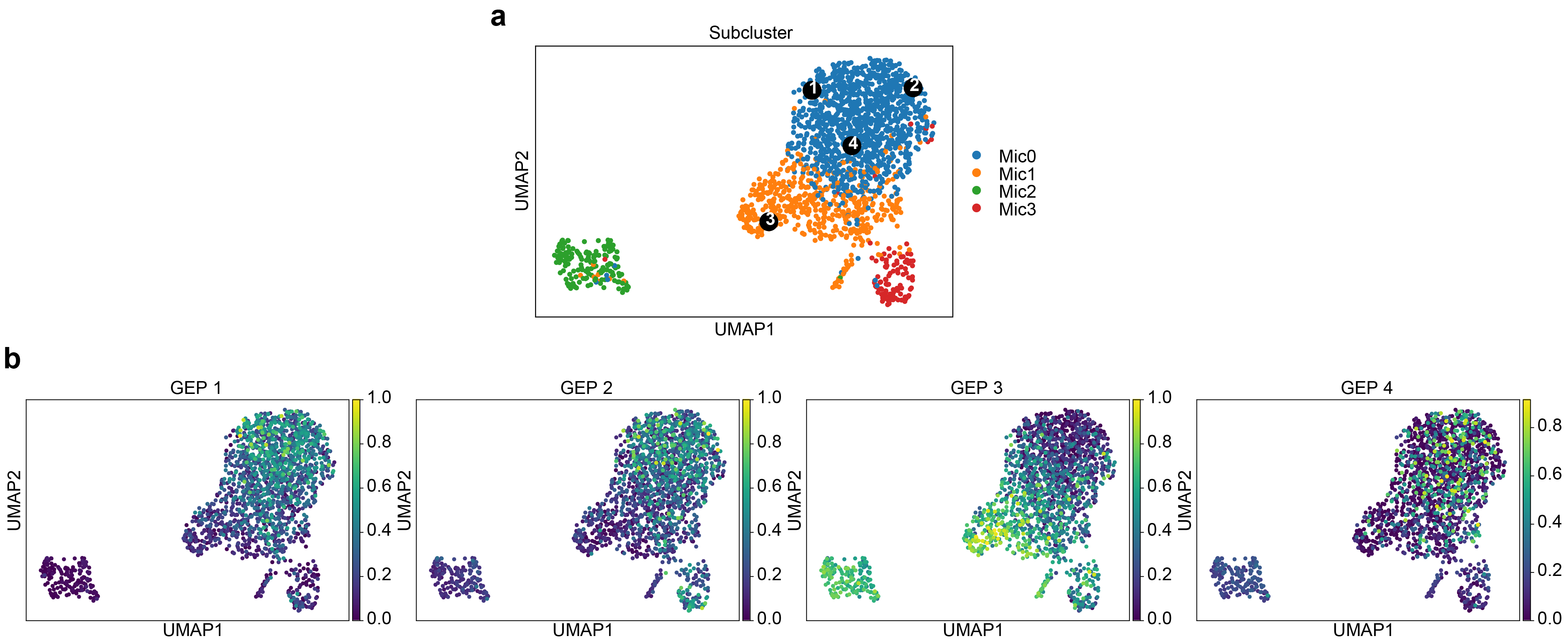

Supplement: S23 Fig — (a) UMAP colored by microglia subclusters. Black dots are locations of cells that have the largest usage of the corresponding GEP (marked in Arabic numerals). (b) UMAPs colored by the inferred usage of each GEP. (TIF) [file pcbi.1010025.s023.tif]

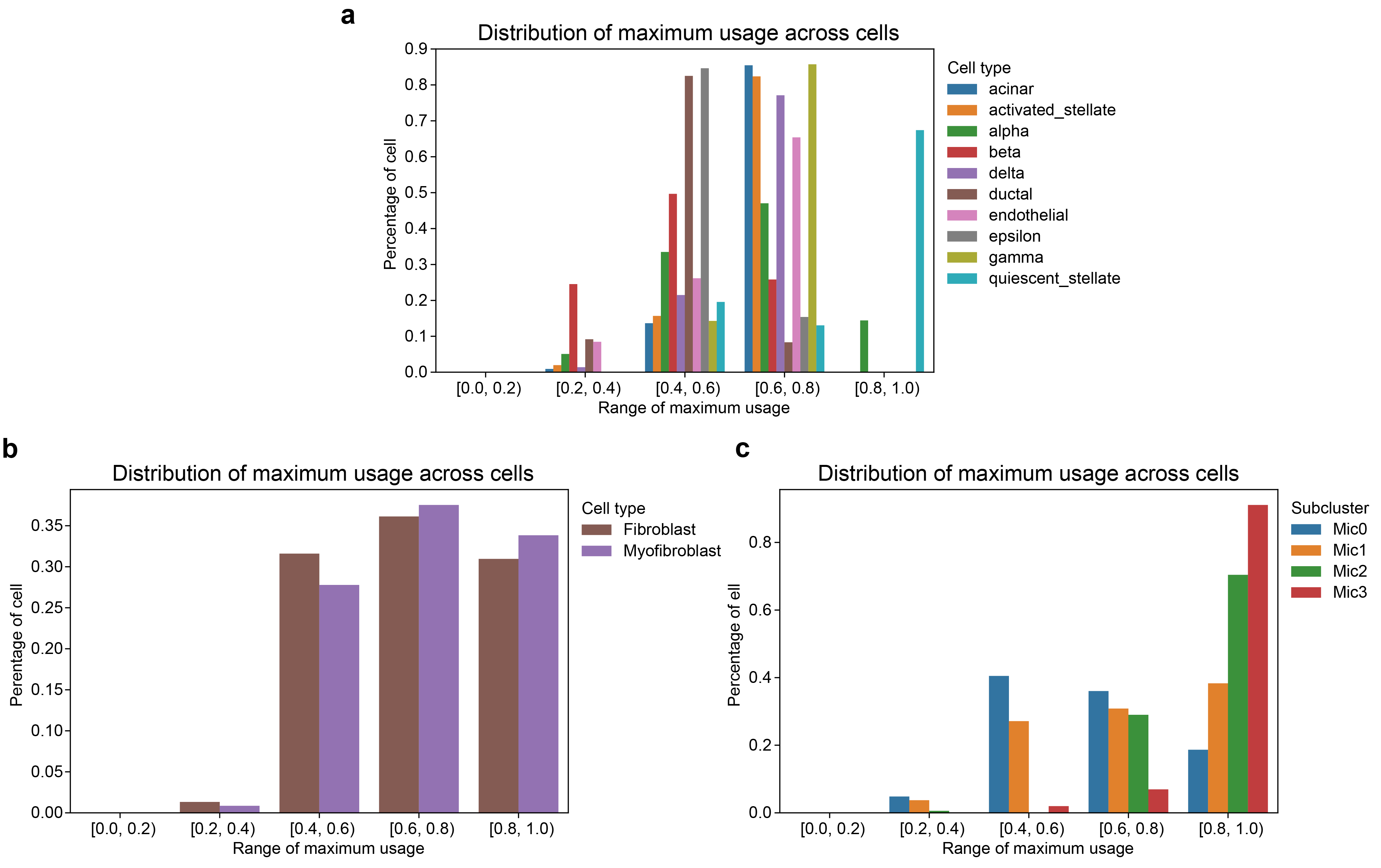

Supplement: S24 Fig — Barplots showing the distribution of the largest inferred usage across cells grouped by annotated clusters in the pancreas islet dataset (a), the lung fibroblasts and myofibroblasts (b), and the microglial cells (c). (TIF) [file pcbi.1010025.s024.tif]

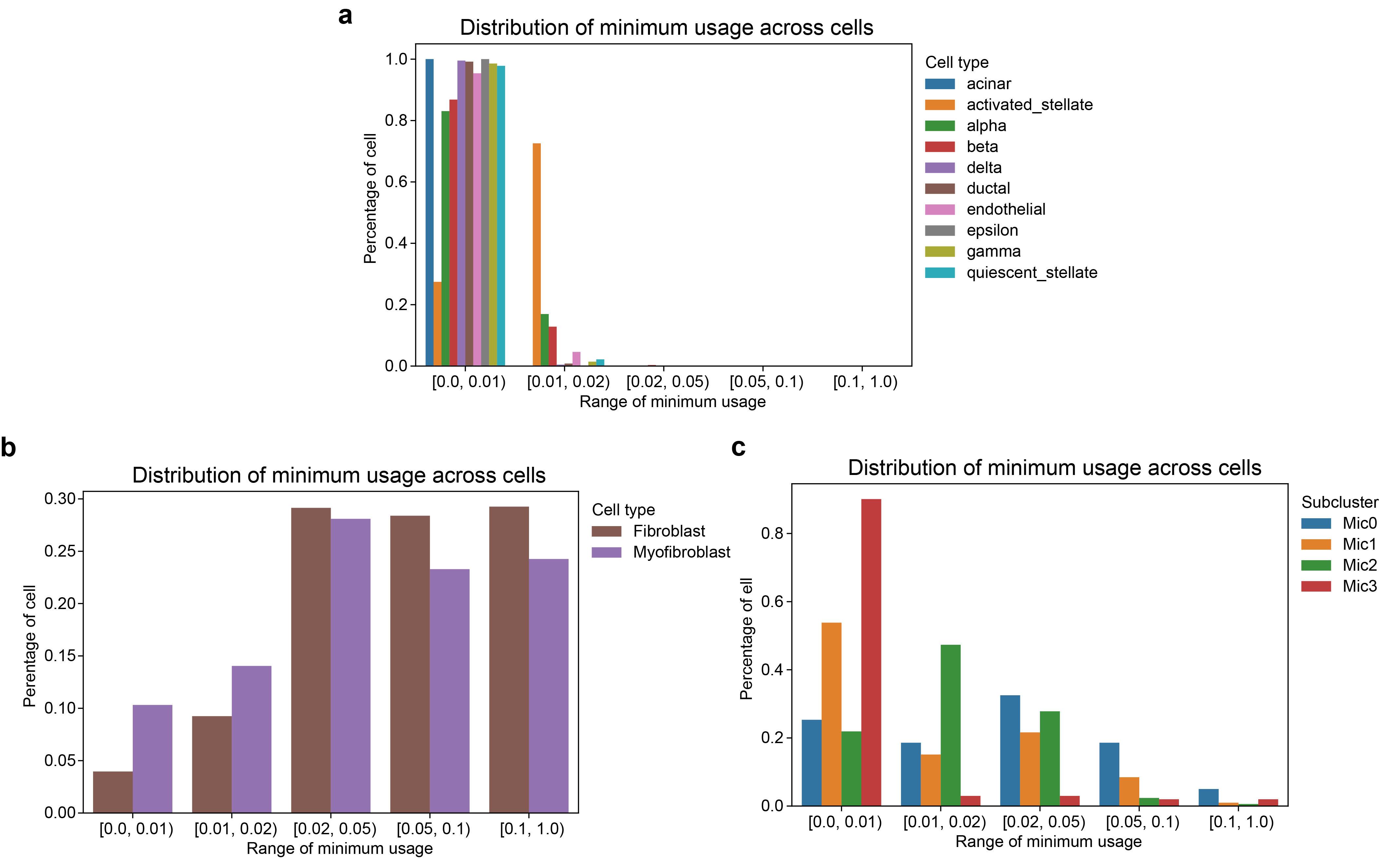

Supplement: S25 Fig — Barplots showing the distribution of the smallest inferred usage across cells grouped by annotated clusters in the pancreas islet dataset (a), the lung fibroblasts and myofibroblasts (b), and the microglial cells (c). (TIF) [file pcbi.1010025.s025.tif]

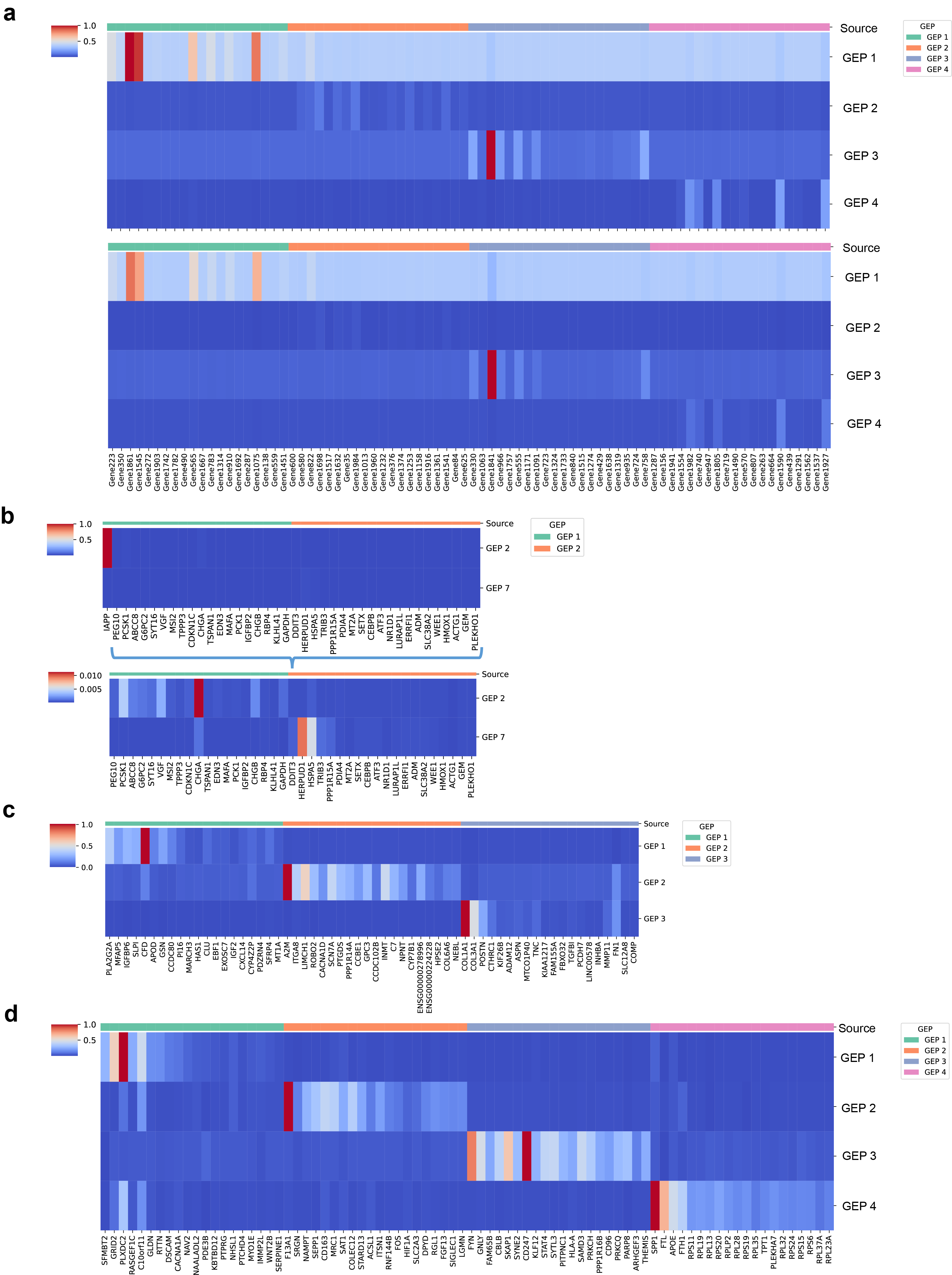

Supplement: S26 Fig — (a) In a simulated dataset, where top is from the true GEP matrix and bottom is from the inferred the GEP matrix (rearranged GEPs to match the order of true GEPs by Pearson correlation); (b) in the pancreatic islet dataset (where the bottom if a subset of the top figure without IAPP for a better resolution); (c) in the lung dataset; (d) in the prefrontal cortex dataset. (TIF) [file pcbi.1010025.s026.tif]
