# Supplementary material for: Non-linear archetypal analysis of single-cell RNA-seq data by deep autoencoders
Source: PLoS Comput Biol. 2022 Apr 1;18(4):e1010025. doi: 10.1371/journal.pcbi.1010025 (PMC9007392; doi:10.1371/journal.pcbi.1010025)
Supplement: S1 Text — (DOCX) [file pcbi.1010025.s054.docx]

**1. Supplementary notes for archetypal analysis and scAAnet**

Linear archetypal analysis has its mathematical definition that was first brought out by Adele Cutler and Leo Breiman in 1994 [4]. For multidimensional data $\boldsymbol{x}_{i=1,\ldots,n}$, where each $\boldsymbol{x}_{i}$ is a vector of length p, the goal of archetypal analysis is to find K archetype vectors $\boldsymbol{z}_{k=1,\ldots,K}$ such that the data can be well approximated by convex combinations of the K archetypes. This is equivalent to minimizing the following loss function

$\sum_{i=1}^{n} \|\boldsymbol{x}_{i}-\sum_{k=1}^{K} a_{ik}\boldsymbol{z}_{k}\|$, where $a_{ik}\geq0$ and $\sum_{k=1}^{K} a_{ik}=1$. (S.1)

However, minimizing equation (S.1) alone does not guarantee that the solution for $\boldsymbol{z}_{k=1,\ldots,K}$ is unique, because we can find coefficient vectors $\boldsymbol{a}_{i=1,\ldots,n}$ making equation (S.1) equal zero as long as $\boldsymbol{z}_{k=1,\ldots,K}$ locate outside the convex hull of the data. Based on the purpose of archetypal analysis, archetypes represent ‘pure’ types in the data and they should resemble the real-world data. Consequently, Adele Cutler and Leo Breiman proposed that archetypes fall on the convex hull of the data so that they are extreme data values. Mathematically, this means

$\boldsymbol{z}_{k}=\sum_{i=1}^{n} b_{ki}\boldsymbol{x}_{i}$, where $b_{ki}\geq0$, $\sum_{i=1}^{n} b_{ki}=1$ and $k=1,\ldots, K$. (S.2)

Note that $\boldsymbol{b}_{k=1,\ldots K}$ is another set of coefficient vectors for archetypes in terms of data values. Minimizers of equation (S.1) satisfying equation (S.2) are archetypes of the data. These two conditions go hand in hand with each other to make sure that the data can be approximated by mixtures of archetypes that are convex combinations of the data values $\boldsymbol{x}_{i=1,\ldots,n}$.

As we discussed in the main text, linear archetypal analysis is not suitable for systems where data values are generated non-linearly from the combination of archetypes, such as scRNA-seq data we focus. A new framework for non-linear archetypal analysis has been proposed based on autoencoders, which has been widely used for non-linear decomposition. The main challenge we face when using autoencoders for archetypal analysis is to consider how to modify the structure of traditional autoencoders to satisfy the two conditions of archetypal analysis in equation (S.1) and (S.2).

The output of the encoder side, matrix $A$, is a latent representation of the data and its row vectors correspond to the coefficient vectors $\boldsymbol{a}_{i=1,\ldots,n}$ in equation (S.1). Therefore, matrix $A$ should be non-negative and have a constant row sum as 1. This constraint can be easily achieved by applying Softmax transformation on the output of the encoder. However, equation (S.2) is harder to satisfy because the non-linear transformation makes the locations of archetypes in the latent space relatively arbitrary. If we take a close look at condition (S.2), we will find what it does is to make inferred archetypes tight to the data. Therefore, one solution is to set the positions of archetypes in the latent space as a priori and penalize the distance between the preset archetypes and inferred archetypes in the latent space. To realize this, we make the encoder not only output the coefficient matrix $A$ but also another coefficient matrix $B^{T}$ whose column vectors correspond to $\boldsymbol{b}_{k=1,\ldots,K}$ in (S.2). Then, we can add an archetypal constraint as a new loss term in equation (3) shown in the main text.

**2. Experiments on the relative weight of the archetypal loss to the reconstruction loss**

To study how the performance of scAAnet would be affected if we changed the relative weight of the archetypal loss, we used 13 different values (0.01, 0.02, 0.05, 0.1, 0.2, 0.5, 1, 2, 5, 10, 20, 50, and 100) as the weight on the archetypal loss and ran scAAnet 13 times with each value on a simulated dataset. From S17 Fig, we can see that the archetypal loss decreased as the weight increased, but the rate of change slowed down as the weight approached 1. In addition, the reconstruction loss and the total loss (reconstruction loss + weight * archetypal loss) did not change much because they were much larger than the archetypal loss in magnitude. For the three metrics assessing the performance of scAAnet in the second row, we can see that the accuracy of cell usage inference was robust to weights around 1, and the performance of scAAnet on GEP inference and reconstruction error were robust to all the chosen values of weight. Therefore, we think 1 is a good choice for weight and set it as scAAnet’s default value.

**3. Robustness of scAAnet to misspecified** $\boldsymbol{K}$

We studied how robust scAAnet was to misspecified K on simulated data. Note that the true number of archetypes in simulated data was 4. We ran scAAnet with K being misspecified at 3 and 4. We also ran scAAnet with correctly specified K at 4. We observed a smooth transition as K increased both quantitively and qualitatively for a simulated dataset in S18 Fig. For example, each of the three GEPs inferred under K=3 had a perfect match with one of the GEPs inferred under K=4 and there were no overlaps among them. Similar phenomenon could be observed when comparing results from K=4 and K=5. Moreover, when K was overspecified (K=5), we can see that GEP 3 inferred under K=4 separated into two GEPs inferred under K=5 (GEP 3 and GEP 4). All the correlation values were larger than 0.97 for those perfectly matched pairs. When K was underspecified (K=3), scAAnet was able to identify a subset of GEPs inferred under the true K for over 80% of the repeated experiments in most simulation settings (S19 Fig). In total, scAAnet was robust to misspecified K around the correct K and the key set of GEPs remained unchanged.

**4. Performance of scAAnet on synthetic data simulated with no shared GEPs**

To further show the generalizability of scAAnet on the extreme case where cells do not share GEPs, we evaluated the performance of scAAnet on data simulated by assigning each cell to only one of the four GEPs. As we can see from the quantitatively results in S20 Fig, scAAnet maintained good performance under this extreme case across different zero inflation rates.

**5. Potential of scAAnet for trajectory analysis**

Another potential usage of scAAnet is for trajectory and pseudo-temporal analysis. Based on the definition of archetypal analysis, we argue that scAAnet can be used to fit developmental data following either a linear trajectory or a bifurcating trajectory. We used two simulated datasets from a benchmarking paper for trajectory inference methods, one with a linear trajectory and the other with a bifurcating trajectory, to showcase the usage of scAAnet on developmental data. We selected the number of archetypes based on the number of milestones in the simulated trajectory data (3 for linear and 4 for bifurcation). As shown in S21 Fig, for both datasets, scAAnet was able to identify GEPs that had good correspondence with known milestones along the trajectories. The closer the cells to a milestone, the more the contribution of the corresponding GEP to the expression profile of the cell. The results indicate the potential of scAAnet for analyzing developmental data with simple trajectory shapes.

**6. Application of NMF to the real data**

To show nonlinearity and count data-based reconstruction loss introduced in scAAnet could help better identify biologically meaningful GEPs, we applied NMF to the pancreatic islet dataset using K = 10 as well. We found NMF delivered noisier results than scAAnet based on UMAP visualization (S22 Fig) similar to Fig 5a in the main manuscript. First, not all cell types had a GEP located on it (e.g. endothelial, ductal, and quiescent stellate cells). Second, these cell types were mixed together in the UMAP generated from the inferred usage matrix or from reconstructed matrix by NMF. These results indicate that NMF was not able to identify all cluster-specific GEP as scAAnet did in this dataset. Similarly, we applied NMF to the microglial cells in the prefrontal cortex dataset with K = 4. Visualization results in S23 Fig showed that NMF failed to identify GEPs that corresponded with the four microglia subclusters. In total, scAAnet had a better performance than NMF on the real data.

**7. The level of shared GEPs in the real data**

We quantified the level of shared GEPs in the three real datasets by first extracting the largest usage value for each cell and binning the values across cells into five windows ([0, 0.2], [0.2, 0.4], [0.4, 0.6], [0.6, 0.8], and [0.8, 1.0]]. From the barplots for each dataset colored by annotated cell clusters in S24 Fig, we can observe that there was a large proportion of cell types in each dataset whose largest cell usage was below 60%. For example, beta and ductal cells in the pancreas islet dataset, fibroblasts and myofibroblasts in the lung dataset, and Mic0 and Mic1 in the prefrontal cortex dataset. Note that for beta cells in the pancreatic islet dataset, since we detected two GEPs there (GEP 2 and GEP 7), the barplot indicates that many beta cells used both GEP 2 and GEP 7 and many of them were not a ‘pure’ GEP 2 or GEP 7-using cell. Overall speaking, the results suggest the phenomenon that many cells contained more than one major GEP and some GEPs were shared across different cell types, so it is reasonable to design an archetypal analysis tool like scAAnet for scRNA-seq studies.

**8. The level of sparsity of inferred usage in the real data**

We quantified the sparsity level of inferred usage in the three real datasets in this section. The smallest usage value for each cell was first obtained. Then the values across cells were binned into five windows ([0, 0.01), [0.01, 0.02), [0.02, 0.05), [0.05, 0.1), and [0.1, 1.0)). As shown in barplots in S25 Fig, we can observe that there existed a non-negligible percentage of cells in each dataset whose smallest usage was below 2% or even below 1%. The percentage was the largest for the pancreas islet dataset where many cell types were categorically delimited. Even for the lung dataset, the percentage of cells with smallest usage below 2% was over 10%. The results suggest the phenomenon that many cells contained only a subset of all the GEPs, so it is reasonable for us to simulate part of the data from the boundary of the simplex.
