# Supplementary material for: Non-linear archetypal analysis of single-cell RNA-seq data by deep autoencoders
Source: PLoS Comput Biol. 2022 Apr 1;18(4):e1010025. doi: 10.1371/journal.pcbi.1010025 (PMC9007392; doi:10.1371/journal.pcbi.1010025)
Supplement: Attachment — Submitted filename: R1.pdf [file pcbi.1010025.s056.pdf]

This manuscript describes another way of decomposing the gene counts as a mixture of GEPs. The output includes a latent, low dimensional representation with a "cell usage" (the "archetypal decomposition") for each cell. The other is a GEP (archetype) matrix that has k GEPs. Similar decompositions include the nonnegative matrix decomposition and the AAnet. The attractive features for scAAnet seem to include that (1) it uses a reconstruction error that is more meaningful (based on negative log likelihood) than MSE (which is essentially NLL assuming Gaussian model) for scRNAseq data (2) it uses a Zfix (preset positions of the archetypes in the latent space) to force the inferred archetypes towards the preset positions.

I like the idea and it is certainly a worthwhile addition to the tools we apply to sc data to make sense of the GEPs. The results are interesting/promising. My main critiques are on the presentation.

Major comments:

1. The organization of the paper can be improved for the readers, as the current presentation is rather confusing and difficult to follow.

    - The supplement text adds more background of the archetype analysis and is very helpful. However, the introduction session does not refer to it. It would have been much easier for a reader unfamiliar with archetype analysis to understand the rest of the paper if they find that supplement early.
    - The Results follow the introduction, as the journal format dictates. But the authors seem to expect that readers would be going back and forth between the Results and the Method sections to make sense of it. It would be much clearer, if the Results section make clearer introduction of the symbols used, though details can be left to the Methods. When it refers to the Methods section, it would help to
        - For example, in Fig 1, it is not clear what the purpose matrix B is for. Fig 1 could also add the key contribution of scAAnet of using a different reconstruction error, which ties the two decoder together. Otherwise it is not clear why one needs two decoders.
        - It is not clear why D2 and D3 are represented as "decoder 2" in Fig 1, instead of three decoders. The Methods section states that "decoder DD_2 and DD_3 also share the same set of layers before their respective output layer"—does it mean all the weight parameters would be the same except the last layer?
        - The captions of the supplementary figures are not with the figures. These are referred to from the main text but little explanation of what is being plotted is provided with the figure. All supplemental figures could use a caption that explains whether the result is based on simulation or which data set is used (for example S2 Fig is on simulation); what is being compared, as these are often not give in detail in the main text. Fig S12 supposedly shows more robust result if the Zfix is trainable. What exactly is shown in the figure? The caption is very short. What reflects robustness? Fig S12 seems to compare different number of warm-up. What is the comparison with not allowing Zfix to be trainable and leave it fixed?

2. Methods:
    - How is Zfix set? In the text it is mentioned that this is "preset" and later saying it is allowed to be trained. But where is it set? This is not explicitly given anywhere. Is it set to be at the vertices of the simplex? Can we visualize a similar plot for the real datasets as Fig 3d?
    - The algorithm is not clear on how the f_D2 and f_D3 are trained though these are supposedly all in the ``update network parameters" step as they all contribute to RE. A little bit of detail here would help. For example, are all sets of parameters (for all

encoders and decoders) updated simultaneously or sequentially? In Eq2, is there a comma missing between μ_i and l_i, or is it meant to be a product?

- Why is the GEP matrix only about the μ (P13 Line 172 explains that the archetypes are obtained b feeding the IZfix to decoder 1), and not the π? It seems that π is just as important, if not more so, than the μ?

3. Results in the simulation:

- Is the data generated with four achetypes and K=4 used for scAAnet? Would you have chosen k=4 if you used the same criteria in the real data to choose k?
- What does λ refer to? It appears in Fig2,Fig 4 and the only text that discusses does not appear until Page 40 , where it is said to "control the dropout ratio"-- but specifically what is it? If these are not going to be referred to in the text, it would help to point to specific sessions where the reader would find answers in. Is there a relationship between λ and pi?
- In measuring the correlation between the true GEPs and the inferred ones – scAAnet has the Zfix to force the archetypes close to the predefined profile, so the inferred archetypes are expected to correspond to the truth, hence the corresponding GEPs would be in the same order of the truth. How is this determined for the other methods?
- MSE is used for both accuracy of cell usage and reconstruction error. I agree that it is not a good choice, so why use it in evaluation? It seems that when scAAnet is using the ZINB loss actually has lower reconstruction MSE (the right column of Fig 2) than the scAAnet that directly aims to minimize the reconstruction MSE. This is counter-intuitive. I am not surprised that scAAnet(ZINB) works better (in a more sensible assessment of error), but I am surprised that it has lower MSE than the scAAnet that directly tries to minimize MSE. What is the explanation here?
- Fig 4 compares AUC – I suppose this is detecting true DEGs that have different expression levels across the k archetypes. Is the DE only introduced in the parameter μ and not pi?

4. Discussion and recommendation:

- The authors stated that scAAnet is best used "on a subset of cell types that are likely to involve shared programs or are of particular interest to researchers". Does this mean that if one has a dataset of multiple cell types, one should first run some clustering or other celltyping, and select a subpopulation?

- Is the only reason against using scAAnet on cell populations involving categorically different types the computation burden?

- Differences between scAAnet with other decomposition includes its nonlinearity (compared to linear methods) and its unique reconstruction error (compared to other deep learning that typically uses MSE as reconstruction error). One group of methods not compared here, that essentially consider the reconstruction error in counting data, but still uses linear decomposition, are methods based on the topic models where the topics have

similar interpretation as the GEPs here. It would be useful to introduce these and discuss their differences.

- It seems that one use of scAAnet would be on data that pseudo-time had been widely estimated. It seems if there is no branching, two archetypes would be sufficient and the cell usage will naturally be between (0,1). If there is a single fork in the pseudo time, three archetypes will be sufficient. Have you considered applying scAAnet to some datasets on which pseudotime appeared to be reasonably successful? Adding an example like that would be really nice. If adding another application is beyond the scope of this work, it would be nice to add some discussion on the anticipated behavior of scAAnet on these datasets.

Minor:

- Preprocessing:

"filtered out genes whose mean expression levels were lower than 0.001" – how is this mean computed? A simple average of raw counts across all cells before any normalization, or average of a library size normalized mean (such as the counts-per-10000 used in the UMAP?)

"We then selected top 2,000 highly variable genes (HVGs) based on the pipeline in Scanpy for scAAnet" – please give the specifics so the readers do not have to look up the definition of HVGs in Scanpy.

What determined the number of the top HVGs in each data set? In Lung it is the top 1500 instead of the top 2000 used in the other two datasets.
