# Supplementary material for: Non-linear archetypal analysis of single-cell RNA-seq data by deep autoencoders
Source: PLoS Comput Biol. 2022 Apr 1;18(4):e1010025. doi: 10.1371/journal.pcbi.1010025 (PMC9007392; doi:10.1371/journal.pcbi.1010025)
Supplement: Attachment — Submitted filename: Response to reviewers.docx [file pcbi.1010025.s057.docx]

**Authors’ Responses**

**Point-by-point responses to the reviewers’ comments:**

Reviewer #1:

This manuscript describes another way of decomposing the gene counts as a mixture of GEPs. The output includes a latent, low dimensional representation with a “cell usage” (the “archetypal decomposition”) for each cell. The other is a GEP (archetype) matrix that has k GEPs. Similar decompositions include the nonnegative matrix decomposition and the AAnet. The attractive features for scAAnet seem to include that (1) it uses a reconstruction error that is more meaningful (based on negative log likelihood) than MSE (which is essentially NLL assuming Gaussian model) for scRNAseq data (2) it uses a Zfix (preset positions of the archetypes in the latent space) to force the inferred archetypes towards the preset positions.

I like the idea and it is certainly a worthwhile addition to the tools we apply to sc data to make sense of the GEPs. The results are interesting/promising. My main critiques are on the presentation.

Major comments:

1. The organization of the paper can be improved for the readers, as the current presentation is rather confusing and difficult to follow.

- The supplement text adds more background of the archetype analysis and is very helpful. However, the introduction session does not refer to it. It would have been much easier for a reader unfamiliar with archetype analysis to understand the rest of the paper if they find that supplement early.

*Response: Thank you for your suggestion. We have added more information in Introduction to refer the readers to supplementary notes about archetypal analysis.*

- The Results follow the introduction, as the journal format dictates. But the authors seem to expect that readers would be going back and forth between the Results and the Method sections to make sense of it. It would be much clearer, if the Results section make clearer introduction of the symbols used, though details can be left to the Methods. When it refers to the Methods section, it would help to
- For example, in Fig 1, it is not clear what the purpose matrix B is for. Fig 1 could also add the key contribution of scAAnet of using a different reconstruction error, which ties the two decoder together. Otherwise it is not clear why one needs two decoders.

*Response: Thank you for your suggestion. We have updated Fig. 1 by explicitly adding the two loss terms (reconstruction loss and archetypal loss) to help emphasize the necessity of matrix B and using three output layers.*

- It is not clear why D2 and D3 are represented as “decoder 2” in Fig 1, instead of three decoders. The Methods section states that “decoder 𝐷𝐷2 and 𝐷𝐷3 also share the same set of layers before their respective output layer”—does it mean all the weight parameters would be the same except the last layer?

*Response: Sorry for the confusion. We have changed notations in Methods (*$E_{1}$*->* $E_{a}$*,* $E_{2}$*->* $E_{b}$*,* $D_{1}$*->* $D_{\mu}$*,* $D_{2}$*->* $D_{\theta}$*,* $D_{3}$*->* $D_{\pi}$*) to make it consistent with notations used in Fig 1. Yes, you are right that for dispersion parameter* $\theta$ *and dropout parameter* $\pi$*, their weight parameters of the decoders are shared except the last layer. This strategy is used in some autoencoder-based methods for scRNA-seq data, such as scVI [1] and deep count autoencoder (DCA) [2].*

- The captions of the supplementary figures are not with the figures. These are referred to from the main text but little explanation of what is being plotted is provided with the figure. All supplemental figures could use a caption that explains whether the result is based on simulation or which data set is used (for example S2 Fig is on simulation); what is being compared, as these are often not give in detail in the main text. Fig S12 supposedly shows more robust result if the Zfix is trainable. What exactly is shown in the figure? The caption is very short. What reflects robustness? Fig S12 seems to compare different number of warm-up. What is the comparison with not allowing Zfix to be trainable and leave it fixed?

*Response: Sorry for the inconvenience. Previously these captions were appended as supporting information at the end of the main text. We have added figure captions below each supplementary figure in the revised manuscript. For the old S12 Fig, we have changed the caption to the following one.*

*‘S14 Fig. Performance of scAAnet over increasing number of warm-up. (a) Cell usage inference performance. MSE between inferred usage and true usage was calculated. (b) GEP inference performance. Pearson correlations between inferred GEPs and true GEPs were calculated. For both a and b, red dashed lines are vertical lines plotted at the number of warm-up periods being 20. Ticks along x-axis are numbers of warm-up periods (0, 5, 10, 20, 30, 40, 50, 70, 100, 150, and 200). The total number of epochs was fixed at 200, so if the number of warm-up period is 200, it means* $Z^{fix}$ *is not allowed to be trainable.’*

2. Methods:

- How is Zfix set? In the text it is mentioned that this is “preset” and later saying it is allowed to be trained. But where is it set? This is not explicitly given anywhere. Is it set to be at the vertices of the simplex? Can we visualize a similar plot for the real datasets as Fig 3d?

*Response: Thank you for your questions. We set* $Z^{fix}$ *based on similar rules used in DeepAA [3]. We have added some details to our manuscript to clarify this point in the first section of Results and modified the last paragraph of Design of scAAnet in Materials and Methods.*

*Results: ‘Basically, each archetype is set with the centroid of the simplex formed by these archetypes is the origin in the latent space and the distances between any two adjacent archetypes are the same.’*

*Materials and Methods: ‘Although the locations of the archetypes in the latent space are set in the beginning, our algorithm allows* $Z^{fix}$ *to be trainable after a warm-up period to make the shape of latent space more flexible to the input data. We found that scAAnet achieved a good balance between the accuracy of cell usage inference and the accuracy of GEP inference on simulated data after a warm-up period of 20 (S14 Fig). After 20, the performance was relatively robust as the number of warm-up periods increased. Therefore, we set the warm-up period to be 20 as the default value and adopt this strategy in real data analysis.’*

*Yes, we have provided MDS visualization for the three real dataset in S6, S10, and S12 Figs, respectively. We also added the following sentences in Results.*

*‘S6 Fig. MDS visualization of archetypal space in the pancreatic islet dataset. (a) MDS visualization colored by cell types. Locations of inferred archetypes are shown as black dots. (b) MDS visualization colored by the inferred usage of the ten GEPs. Locations of inferred archetypes are shown as red dots.’*

*‘S10 Fig. MDS visualization of archetypal space in the lung fibroblast and myofibroblast cells. (a) MDS visualization colored by cell types. (b) MDS visualization colored by disease groups. Locations of inferred archetypes in a and b are shown as black dots. (c) MDS visualization colored by the inferred usage of the three GEPs. Locations of inferred archetypes are shown as red dots.’*

*‘S12 Fig. MDS visualization of archetypal space of microglial cells in the prefrontal cortex dataset. (a) MDS visualization colored by subclusters. (b) MDS visualization colored by AD pathology groups. (c) MDS visualization colored by sexes. Locations of inferred archetypes in a, b and c are shown as black dots. (d) MDS visualization colored by the inferred usage of the four GEPs. Locations of inferred archetypes are shown as red dots.’*

*‘As shown in the two-dimensional MDS plots based on results from scAAnet (S6 Fig), the inferred archetypes were tight to the data in the archetypal space.’*

*‘From MDS visualization (S12 Fig), we can observe that each microglia subcluster was enriched at the corresponding corner of the simplex.’*

- The algorithm is not clear on how the f_D2 and f_D3 are trained though these are supposedly all in the “update network parameters” step as they all contribute to RE. A little bit of detail here would help. For example, are all sets of parameters (for all encoders and decoders) updated simultaneously or sequentially? In Eq2, is there a comma missing between 𝜇𝑖 and 𝑙𝑖, or is it meant to be a product?

*Response: Thank you for your comments. The parameters of the entire network are updated simultaneously through backpropagation. We have added some details including backpropagation in Algorithm 1. In Eq2, it should be* $\mu_{i}$ *times* $l_{i}$*, where* $\mu_{i}$ *is a vector and* $l_{i}$ *is a scalar, so there is no symbol missing.*

- Why is the GEP matrix only about the $\mu$ (P13 Line 172 explains that the archetypes are obtained by feeding the IZfix to decoder 1), and not the $\pi$? It seems that $\pi$ is just as important, if not more so, than the $\mu$?

*Response: Thank you for your question. Here, we only consider the mean parameter* $\mu$ *for the inference of GEP matrix but not the dropout parameter* $\pi$ *because the dropout is usually regarded as noise in scRNA-seq data. Similar methods (scVI [1], DCA [2], and scImpute [4]) based on ZINB distributions or other zero-inflated distributions also only use inferred mean vectors to perform downstream analysis.*

3. Results in the simulation:

- Is the data generated with four archetypes and K=4 used for scAAnet? Would you have chosen k=4 if you used the same criteria in the real data to choose k?

*Response: Thank you for the question. The answer is yes to both questions. If we used the same criteria as those used in the real data, K would also be chosen as 4 in simulated data. We have picked one simulated data, presented results in S1 Fig and added the following sentences to mention this point.*

*‘S1 Fig. Selection of K in a simulated dataset. (a) Stability of usage, (b) stability of archetypes and (c) reconstruction loss across different Ks for four count distributions. (d) Distance matrix showing archetype stability under K=2-5. The darker the color the smaller the distance. (e) Consensus clustering matrices showing the usage stability under K = 2-5. The warmer the color the large the similarity.’*

*‘We used* $K=4$ *to train scAAnet on simulated data. Note that in real data, it would be impossible for us to know the true number of archetypes and we propose to select* $K$ *based on the stability of both inferred usage and GEP matrices (Methods). As shown in S1 Fig, we were able to select* $K=4$ *for simulated data after considering model stability in general.’*

- What does 𝜆 refer to? It appears in Fig2,Fig 4 and the only text that discusses does not appear until Page 40 , where it is said to “control the dropout ratio”-- but specifically what is it? If these are not going to be referred to in the text, it would help to point to specific sessions where the reader would find answers in. Is there a relationship between 𝜆 and pi?

*Response: Thank you for pointing this out. 𝜆 controls the level of dropout. When it becomes smaller, the probability of dropout (*$\pi$*) would decrease given the same values of mean expression levels drawn from Gamma distributions. We have added the following statements in Results and Methods to clarify this.*

*Results: ‘where* $\lambda$ *is a parameter used in the simulation process to introduce dropout (Methods).’*

*Methods: ‘For cell* $i$ *and gene* $j$*, its dropout probability is defined as*
$\pi_{ij}=exp(-\lambda\mu_{\mathrm{ij}}^{2}),$ *where* $\mu_{ij}$ *is the mean expression level drawn from a Gamma distribution and such a relationship was used in a previous scRNA-seq study [5].’*

- In measuring the correlation between the true GEPs and the inferred ones – scAAnet has the Zfix to force the archetypes close to the predefined profile, so the inferred archetypes are expected to correspond to the truth, hence the corresponding GEPs would be in the same order of the truth. How is this determined for the other methods?

*Response: Thank you for the question. First, we would like to clarify that* $Z^{fix}$ *does not absolutely guarantee that the inferred archetypes are close to the underlying true archetypes in the original feature space. It only ensures that the archetypes in the latent space are tight to the data. Given the same number of archetypes K, the preset* $Z^{fix}$ *would be the same for different input data, which means that it only depends on K. Second, to answer your question about how we matched inferred GEPs to true GEPs in order to quantify the accuracy of GEP inference, we calculated the correlation between any pairs of inferred GEP and true GEP vector and for each inferred GEP, we paired it with the true GEP with the highest correlation. If two inferred GEPs were paired to the same true GEP, whichever with larger correlation would be paired with the true GEP and the other one would be paired with the true GEP whose correlation was the second largest. Pairs determined by GEP vectors would also be used for quantifying the accuracy of usage vector inference.*

- MSE is used for both accuracy of cell usage and reconstruction error. I agree that it is not a good choice, so why use it in evaluation? It seems that when scAAnet is using the ZINB loss actually has lower reconstruction MSE (the right column of Fig 2) than the scAAnet that directly aims to minimize the reconstruction MSE. This is counter-intuitive. I am not surprised that scAAnet (ZINB) works better (in a more sensible assessment of error), but I am surprised that it has lower MSE than the scAAnet that directly tries to minimize MSE. What is the explanation here?

*Response: Although MSE is not a good metric to measure the reconstruction error for count data, we still use it to quantify the reconstruction accuracy because the reconstruction MSE is calculated between the reconstructed mean and the true mean expression levels. Note that the reconstructed matrix is obtained from the output layer for* $\mu$*, which is the mean parameter of the count distribution. Therefore, we didn’t compare it with the simulated raw count data, but with the underlying mean of the data. Also note that scAAnet with MSE loss aims to minimizes MSE between input raw count data and the reconstructed mean, but not the MSE between the true mean and the reconstructed mean. However, scAAnet with ZINB loss has a distribution assumption closer to the underlying truth. Therefore, it is reasonable for scAAnet (ZINB) to have lower reconstruction MSE compared with scAAnet (MSE). To aid future readers on this point, we have added the following statement to our manuscript in Results.*

*‘where reconstruction errors were calculated between the inferred scaled mean and the true scaled mean expression levels’*

- Fig 4 compares AUC – I suppose this is detecting true DEGs that have different expression levels across the k archetypes. Is the DE only introduced in the parameter $\mu$ and not $pi$?

*Response: Thank you for your question. To clarify, when simulating data with 4 archetypes/GEPs from ZINB distributions, DEGs were introduced first to archetype vectors where DEGs were randomly selected for each archetype. Then we simulated usage vector for each cell from Dirichlet distributions. The mean gene expression vector for each cell* $i$ *(*$\mu_{i}$*) was calculated by multiplying the usage vector by the GEP matrix. The dropout parameter for cell* $i$ *(*$\pi_{i}$*) was determined based on the mean expression vector. So, to some extent, DEGs would affect both* $\mu$ *and* $\pi$*. Another clarification is that DEGs were detected based on inferred usage vectors by regressing the observed raw count expression data of each gene on each of the inferred usage vector. A gene with significant results on a usage vector would be recognized as a DEG for the corresponding GEP. We didn’t directly regard top genes in the inferred GEP vector as DEGs because there may be shared top genes across GEPs.*

4. Discussion and recommendation:

- The authors stated that scAAnet is best used “on a subset of cell types that are likely to involve shared programs or are of particular interest to researchers”. Does this mean that if one has a dataset of multiple cell types, one should first run some clustering or other celltyping, and select a subpopulation?

*Response: Thank you for your question. Generally speaking, running some clustering before scAAnet is recommended, but this is not a strict rule, because one can always run scAAnet using the entire dataset and pick interesting archetypes/GEPs for further analyses. As shown in the pancreas islet dataset, either using the entire dataset or only beta cells, we could always find the same two GEPs underlying beta cells. However, we do suggest users to run scAAnet on subpopulations where cell types are not clearly separated (one can decide this based on UMAP visualization), indicating potential shared expression programs, in order to save time and computing resources. One can also divide data into several relatively large groups of cell populations based on domain knowledge about the relationship between main cell types. For example, scAAnet can be run on lymphocytes or monocytes and macrophages separately. Choosing to run scAAnet on which level of cell population is a decision based on prior knowledge, visualization, and pre-clustering together. We have extended the following sentence in Discussion.*

*‘we recommend users to run scAAnet on a subset of cell types that are likely to involve shared programs (UMAP visualization and pre-clustering combined with domain knowledge can be used to determine this)’*

- Is the only reason against using scAAnet on cell populations involving categorically different types the computation burden? Differences between scAAnet with other decomposition includes its nonlinearity (compared to linear methods) and its unique reconstruction error (compared to other deep learning that typically uses MSE as reconstruction error). One group of methods not compared here, that essentially consider the reconstruction error in counting data, but still uses linear decomposition, are methods based on the topic models where the topics have similar interpretation as the GEPs here. It would be useful to introduce these and discuss their differences.

*Response: Thank you for your question and suggestion. The short answer to your question is yes. To some degree, we think scAAnet can be used as an alternative for traditional clustering-based methods when data are purely categorically delimited. As shown in the pancreas islet dataset where cell types are clearly defined and visually separated, scAAnet could find archetypes that have obvious correspondence with pre-annotated cell types. However, if all cell clusters are well defined and there is no indication of potential sharing of GEPs across cell types, it would be an overkill to apply scAAnet. However, it never hurts to run the methods, and the more important point is on which scale of cell populations to run it.*

*Based on your suggestion, we have mentioned topic models in the Discussion section and compared differences and similarities between scAAnet and topic models.*

*‘It is worth mentioning that if we replace all non-linear activation functions by identity functions and remove the hidden layer in all decoders/encoders, scAAnet would perform linear archetypal decomposition on count data. This is equivalent to one group of methods based on topic models [6, 7], such as latent semantic indexing [8] and latent Dirichlet allocation [9].’*

- It seems that one use of scAAnet would be on data that pseudo-time had been widely estimated. It seems if there is no branching, two archetypes would be sufficient and the cell usage will naturally be between (0,1). If there is a single fork in the pseudo time, three archetypes will be sufficient. Have you considered applying scAAnet to some datasets on which pseudotime appeared to be reasonably successful? Adding an example like that would be really nice. If adding another application is beyond the scope of this work, it would be nice to add some discussion on the anticipated behavior of scAAnet on these datasets.

*Response: Thank you for your comments. Trajectory/pseudo-temporal analysis is a very popular topic in single-cell data analysis. In the original paper of the lung dataset considered in our manuscript, the authors analyzed the same group of cells (fibroblasts and myofibroblasts) using pseudotime-based techniques, which was not discussed in our original manuscript. We have added the following statements to the lung data section in Results to point readers to this.*

*‘Note that in the original paper of this dataset, two pseudotime-based archetypes were identified within myofibroblasts and fibroblasts separately, and myofibroblasts ordered towards the end of the trajectory were found to be enriched in IPF lungs. Therefore, scAAnet was not only able to recover the trajectory-based archetype that was both IPF and myofibroblast enriched, but also identify one more archetype in fibroblasts, suggesting the potential of scAAnet on trajectory-based analysis at a higher resolution.’*

*Additionally, we have used data simulated under linear and bifurcation trajectories and showed scAAnet could identify GEPs corresponding to milestones alongside the simulated trajectories. We have added the following results and sentences about the potential of scAAnet on developmental data in S21 Fig, Results, S5 Text, and Discussion.*

*‘S21 Fig. scAAnet identified GEPs corresponding to milestones in simulated trajectory-based data. (a) Linear trajectory with three milestones colored by inferred GEP usage. (b) Bifurcation trajectory with four milestones colored by inferred GEP usage. Milestones are marked as black dots and backbone of the trajectory is shown in black curves.’*

*Results: ‘We explored the potential of scAAnet … and on developmental data simulated under linear and bifurcating trajectories in S5 Text.’*

*S5 Text: ’Another potential usage of scAAnet is for trajectory and pseudo-temporal analysis. Based on the definition of archetypal analysis, we argue that scAAnet can be used to fit developmental data following either a linear trajectory or a bifurcating trajectory. We used two simulated datasets from a benchmarking paper for trajectory inference methods, one with a linear trajectory and the other with a bifurcating trajectory, to showcase the usage of scAAnet on developmental data. We selected the number of archetypes based on the number of milestones in the simulated trajectory data (3 for linear and 4 for bifurcation). As shown in S21 Fig, for both datasets, scAAnet was able to identify GEPs that had good correspondence with known milestones along the trajectories. The closer the cells to a milestone, the more the contribution of the corresponding GEP to the expression profile of the cell. The results indicate the potential of scAAnet for analyzing developmental data with simple trajectory shapes.’*

*Discussion: ‘Fourth, although we have shown the potential of scAAnet on trajectory analysis when the topology is simple (e.g. linear or bifurcation), scAAnet cannot replace state-of-the-art trajectory inference methods because most developmental data contain a more complex topology (e.g. multifurcation or circular) [10]. Encompassing such data within a simplex would result in loss of resolution. Therefore, we recommend using scAAnet as a complement for trajectory analysis when the topology is simple, or data are measured within a relatively short developmental period.’*

Minor:

- Preprocessing:

“filtered out genes whose mean expression levels were lower than 0.001” – how is this mean computed? A simple average of raw counts across all cells before any normalization, or average of a library size normalized mean (such as the counts-per-10000 used in the UMAP?)

*Response: Thank you for your questions. The mean is calculated based on raw counts before any normalization. We have added the following statement below to Methods to clarify this point.*

*‘We filtered out genes with non-zero expression in fewer than 3 cells and genes whose mean expression levels were lower than 0.001 across all cells before normalization.’*

“We then selected top 2,000 highly variable genes (HVGs) based on the pipeline in Scanpy for scAAnet” – please give the specifics so the readers do not have to look up the definition of HVGs in Scanpy.

*Response: Thank you for your suggestion. We have added the following sentence to Methods.*

*‘HVGs were selected by first modeling the relationship between mean and dispersion of all genes across cells. Genes with higher dispersion compared to genes with similar mean expression would be regarded as HVGs [11].’*

- What determined the number of the top HVGs in each data set? In Lung it is the top 1500 instead of the top 2000 used in the other two datasets.

*Response: Thank you for your question. To determine the number of the top HVGs in each dataset, we first referred to the original paper to see the number used by the authors. For example, for the lung dataset, the authors used the top 1,500 HVGs for fibroblasts and myofibroblast to perform all the downstream analyses, so we also used this number. For datasets without clearly clarified number of HVGs in their original paper, we used 2,000, which is the default number used in most scRNA-seq studies. We have added explanation about the special choice of top 1,500 HVGs for the lung data in Methods.*

*‘We then selected top 1,500 HVGs from this dataset for analysis according to the original paper.’*

Reviewer #2:

In this manuscript, Wang and Zhao propose scAAnet, a deep autoencoder for non-linear archetypal analysis of single-cell RNA-seq data. Building on the assumption that the expression profile of each cell results from a non-linear combination of multiple gene expression programs (GEPs), scAAnet aims to detect GEPs and infer the relative activity of each GEP across cells. There are many merits in this work. The manuscript is well organized and clearly written. The methods are innovative and clearly presented, based on conceptually reasonable assumptions. The simulations were carefully designed and novel biological insights revealed from applications to several real datasets.

I have two relatively major comments. First, scAAnet assumes that cell types share some GEPs. While this is conceptually justifiable, can the authors quantify the level of shared GEPs in real data, or evaluate how would scAAnet perform if the cells don’t share GEPs in the extreme case?

*Response: Thank you for your suggestions. We have generated S24 Fig to show the level of sharing in the real data and added S7 Text for discussions on the results.*

*‘S24 Fig. The level of sharing of GEPs in the real data. Barplots showing the distribution of the largest inferred usage across cells grouped by annotated clusters in the pancreas islet dataset (a), the lung fibroblasts and myofibroblasts (b), and the microglial cells (c).’*

*‘We quantified the level of shared GEPs in the three real datasets by first extracting the largest usage value for each cell and binning the values across cells into five windows ([0, 0.2), [0.2, 0.4), [0.4, 0.6), [0.6, 0.8), and [0.8, 1.0)). From the barplots for each dataset colored by annotated cell clusters in S24 Fig, we can observe that there was a large proportion of cell types in each dataset whose largest cell usage was below 60%. For example, beta and ductal cells in the pancreas islet dataset, fibroblasts and myofibroblasts in the lung dataset, and Mic0 and Mic1 in the prefrontal cortex dataset. The results suggest the phenomenon that many cells contained more than one major GEP and some GEPs were shared across different cell types, so it is reasonable to design an archetypal analysis tool like scAAnet for scRNA-seq studies.’*

*To evaluate how scAAnet performs if the cells don’t share GEPs in the extreme case, we have added results under this assumption in S20 Fig and put the following sentences in Results and S4 Text.*

*‘S20 Fig. Performance of scAAnet and other methods on datasets simulated with no shared GEPs. (a) Results from datasets that were simulated under NB distributions. (b) Results under ZINB distributions with* $\lambda$ *= 0.3. (c) Results under ZINB distributions with* $\lambda$ *= 0.1. (d) Results under ZINB distributions with* $\lambda$ *= 0.01. Other details are the same as S2 Fig.’*

*Results: ‘We explored the potential of scAAnet on data simulated purely from one GEP in S4 Text’*

*S4 Text: ‘To further show the generalizability of scAAnet on the extreme case where cells do not share GEPs, we evaluated the performance of scAAnet on data simulated by assigning each cell to only one of the four GEPs. As we can see from the quantitatively results in S20 Fig, scAAnet maintains good performance under this extreme case across different zero inflation rates.’*

Second, scAAnet needs to preset archetype. It is unclear how to preset the values. How would scAAnet’s performance vary with different hyperparameter choices. In the pseudo codes, there seems a warm up period where the Z^fix will be updated simultaneously. However, if that is the case, it seems that equation (3) on page 32 is no longer identifiable?

*Response: Thank you for your comments. We set* $Z^{fix}$ *based on similar rules used in DeepAA. We have added some details to our manuscript in the first section of Results.*

*‘Basically, each archetype is set with the centroid of the simplex formed by these archetypes is the origin in the latent space and the distances between any two adjacent archetypes are the same.’*

*There are two main hyperparameters for scAAnet, one is the number of warm-up periods, and the other is the weight of the archetypal loss. We have modified the following paragraph in Materials and Methods to give a clearer explanation on this parameter.*

*‘Although the locations of the archetypes in the latent space are set in the beginning, our algorithm allows* $Z^{fix}$ *to be trainable after a warm-up period to make the shape of latent space more flexible to the input data. We found that scAAnet achieved a good balance between the accuracy of cell usage inference and the accuracy of GEP inference on simulated data after a warm-up period of 20 (S14 Fig). After 20, the performance was relatively robust as the number of warm-up period increased. Therefore, we set the warm-up period to be 20 as the default value and adopt this strategy in real data analysis.’*

*In addition, we have added one more experiment to test the performance of scAAnet across different weights of the archetypal loss (S17 Fig). The following paragraphs have also been added to Results and S2 Text separately.*

*‘S17 Fig. Influence of relative weight of the archetypal loss on scAAnet. (a) Change of the archetypal loss. (b) Change of the reconstruction loss. (c) Change of the total loss. (d) Performance on cell usage inference across different weights. (e) Performance on GEP inference across different weights. (f) Performance on reconstruction accuracy across different weights.’*

*Results: ‘The total loss of scAAnet is composed of both the archetypal loss and the reconstruction loss and one can change the relative weight of the two losses. Through simulation studies we found that the performance of scAAnet is robust to the change of the weight around 1, so we set the relative weight of the archetypal loss as 1 by default (see S2 Text for more discussions).’*

*S2 Text: ‘To study how the performance of scAAnet would be affected if we changed the relative weight of the archetypal loss, we used 13 different values (0.01, 0.02, 0.05, 0.1, 0.2, 0.5, 1, 2, 5, 10, 20, 50, and 100) as the weight on the archetypal loss and ran scAAnet 13 times with each value on a simulated dataset. From S17 Fig, we can see that the archetypal loss decreased as the weight increased, but the rate of change slowed down as the weight approached 1. In addition, the reconstruction loss and the total loss (reconstruction loss + weight * archetypal loss) did not change much because they were much larger than the archetypal loss in magnitude. For the three metrics assessing the performance of scAAnet in the second row, we can see that the accuracy of cell usage inference was robust to weights around 1, and the performance of scAAnet on GEP inference and reconstruction error were robust to all the chosen values of weight. Therefore, we think 1 is a good choice for weight and set it as scAAnet’s default value.’*

*Although Eq(3) is not identifiable after the warm-up period, since scAAnet is trained through backpropagation and* $Z^{fix}$*is realized through a layer after the layers generating* $A$ *and* $B$*,* $Z^{fix}$*will be updated at first in each iteration and issues won’t occur in this manner. As mentioned above in S17 Fig, warm-up periods longer than 20 all delivered relatively good performance. This demonstrate if we fixed* $Z^{fix}$*for enough number of iterations, identifiability wouldn’t be a problem.*

I have some additional minor comments.

1. Can GEPs be used to infer cell types? Would it be possible to use the inferred mixture of GEPs to infer cell type?

*Response: We think GEPs can be used as a complement for cell type annotation. For example, if marker genes of a known cell type are enriched in the top genes of a GEP, cells with larger usage of the GEP may be annotated as the corresponding cell type. More importantly, if more than one GEP, which are known to be associated with some cell types, dominate a cell population, it indicates that cell population may be a new cell type that has properties from multiple known cell types.*

2. GEPs identified in real data show their values in revealing underlying latent factors, pathways or network. I assume other scRNA-seq methods can also be used for similar purposes, in more convoluted manners, for example, by first clustering the cells and then performing analysis based on cell type differentially expressed genes? Presumably, without the meaningful reduction into GEPs, alternative methods that do not summarize data into GEPs would provide much noisier conclusions? The authors can consider trying competing methods on some of their real datasets.

*Response: Thank you for your comments and questions. For the lung fibroblasts and myofibroblasts dataset, we identified three GEPs with two corresponding to the two cell types and one specific to myofibroblasts from IPF patients. If we perform traditional cluster analysis or cell annotations and cluster based DEG tests, we would only be able to identify DEGs specific to the two cell types. However, we would not be able to find the myofibroblast-and-IPF-enriched GEP at the same time. Even though it is possible for us to find DEGs related to this special GEP by deliberately comparing myofibroblasts from IPF patients with others, we cannot quantify the relative usage of this GEP across cells through cluster-based analysis. In total, we think summarizing data into GEPs would be beneficial for both identifying GEPs at a higher level without predefined categorical clusters in an unsupervised manner and quantifying the contribution of GEPs to different cells.*

3. The authors showed that shared pattern across cell types/disease can be obtained from inferred GEPs. But we can also analyze the cell type marker genes/ disease marker genes and compare the similarity of marker genes in different cell types/ diseases. What will be the benefit of scAAnet if the GEP analysis eventually goes back to GEP marker gene analysis?

*Response: Thank you for your question. Let’s focus on cell types for simplicity. scAAnet is not only able to find GEPs shared across cell types, but also able to identify cell types with more than one active GEP. The former property might be replaced by the cluster-based mark gene analysis you described, but the latter advantage is unique to GEP-based analysis. For example, in the prefrontal cortex dataset, both GEP 2 and GEP 4 were active in microglia subcluster Mic1 (Fig 9f, 9g). Their relationship with AD pathology group suggests that the known association between Mic1 and AD may be positively related to GEP 4 but negatively related to GEP 2.*

Reviewer #3:

The paper proposes an interesting model for understanding the gene expression programs underlying cells from a population with continuous cell states. The nonnegativity and convexity required in archetypal decomposition enable ease of interpretation. Compared with linear archetypal decomposition by NMF, scAAnet incorporates nonlinearity naturally through an autoencoder.

- The paper has discussed several conceptual advantages of scAAnet including nonlinearity and the inclusion of the archetypal constraint. While it is clear the method achieves better results on the simulated data, I was wondering if some comparison can be performed on any of the real data examples, in particular with NMF based methods. Does the nonlinearity allow scAAnet to capture different biological signals? Some comparison will help practitioners better understand the utility of the method, given scAAnet comes with additional computational cost.

*Response: Thank you for your comments. We have added visualization results by applying NMF to the pancreas dataset as S22 Fig and to the microglia subcluster dataset as S23 Fig. We also have added the following sentence and paragraph to Results and S6 Text.*

*‘S22 Fig. UMAP visualization using NMF results on the pancreatic islet dataset. From left to right are UMAPs plotted using the top 35 PCs of the observed scRNA-seq data, using the inferred cell usage matrix, and using the reconstructed expression matrix. UMAPs are colored by 10 known cell clusters. Black dots are locations of cells that have the largest usage of the corresponding GEP (marked in Arabic numerals).’*

*‘S23 Fig. UMAP visualization using NMF results on microglial cells in the prefrontal cortex dataset. (a) UMAP colored by microglia subclusters. Black dots are locations of cells that have the largest usage of the corresponding GEP (marked in Arabic numerals). (b) UMAPs colored by the inferred usage of each GEP.’*

*Results: ‘We also applied NMF to this dataset using K = 10 for comparison. However, results were not satisfactory (see S6 Text for more discussion).’*

*S6 Text: ‘To show nonlinearity and count data-based reconstruction loss introduced in scAAnet could help better identify biologically meaningful GEPs, we applied NMF to the pancreatic islet dataset using K = 10 as well. We found NMF delivered noisier results than scAAnet based on UMAP visualization (S22 Fig) similar to Fig 5a in the main manuscript. First, not all cell types had a GEP located on it (e.g. endothelial, ductal, and quiescent stellate cells). Second, these cell types were mixed together in the UMAP generated from the inferred usage matrix or from reconstructed matrix by NMF. These results indicate that NMF was not able to identify all cluster-specific GEP as scAAnet does in this dataset. Similarly, we applied NMF to the microglial cells in the prefrontal cortex dataset with K = 4. Visualization results in S23 Fig showed that NMF failed to identify GEPs that corresponded with the four microglia subclusters. In total, scAAnet had a better performance than NMF on the real data.’*

- The paper has explained how to choose K, the number of archetypes, through stability criteria. How robust is the method to actually misspecified K? It is a point worth investigating at least on simulated data.

*Response: Thank you for your question. We have investigated the robustness of the method to misspecified K on simulated data by running scAAnet with K being 3, 4, and 5 respectively and drawing correlation heatmaps among inferred GEPs. Results are summarized in S18 Fig. and S3 Text. We also added the following sentence in Results to point readers to S3 Text.*

*‘S18 Fig. Robustness of scAAnet to misspecified K in a simulated dataset. (a) Correlation heatmap between pairs of archetypes inferred using K=3 and K=4 (left), and between inferred using K=5 and K=4 (right). (b) UMAPs colored by the inferred GEP usage. From top to bottom are results based on K being 3, 4, and 5, respectively. Orange arrows indicate the progression of GEPs as K increases.’*

*‘S19 Fig. Robustness of scAAnet to misspecified K across all simulation settings. Each bar represents a simulation setting where ten repeated experiments were run under different seeds. The percentage on the y-axis was calculated as the number of experiments where GEPs inferred under K=3 constituted a perfect subset of GEPs inferred under K=4 divided by ten. A perfect subset was defined as no overlaps among the GEPs inferred under K=4 that had the largest correlations with the GEPs inferred under K=3.’*

*Results: ‘We also explored the robustness of scAAnet to misspecified* $K$*in S3 Text.’*

*S3 Text: ‘We studied how robust scAAnet was to misspecified K on simulated data. Note that the true number of archetypes in simulated data was 4. We ran scAAnet with K being misspecified at 3 and 4. We also ran scAAnet with correctly specified K at 4. We observed a smooth transition as K increased both quantitively and qualitatively for a simulated dataset in S18 Fig. For example, each of the three GEPs inferred under K=3 had a perfect match with one of the GEPs inferred under K=4 and there were no overlaps among them. Similar phenomenon could be observed when comparing results from K=4 and K=5. Moreover, when K was overspecified (K=5), we can see that GEP 3 inferred under K=4 separated into two GEPs inferred under K=5 (GEP 3 and GEP 4). All the correlation values were larger than 0.97 for those perfectly matched pairs. When K was underspecified (K=3), scAAnet was able to identify a subset of GEPs inferred under the true K for over 80% of the repeated experiments in most simulation settings (S19 Fig). In total, scAAnet was robust to misspecified K around the correct K and the key set of GEPs remained unchanged.’*

- The archetypal constraint assumes all cells lie in a tight convex hull in the latent space, which seems a bit restrictive to me. For example, many developmental data have trajectories with bifurcation structure. It would be great if the authors could provide more discussion on the feasibility of this assumption.

*Response: Thank you for your comments. Trajectory/pseudo-temporal analysis is a very popular topic in single-cell data analysis. In the original paper of the lung dataset we used, the authors analyzed the same group of cells (fibroblasts and myofibroblasts) using pseudotime-based techniques, which we didn’t point out in our manuscript. We have added the following sentences to the lung data section in Results to point readers to this.*

*‘Note that in the original paper of this dataset, two pseudotime-based archetypes were identified within myofibroblasts and fibroblasts separately, and myofibroblasts ordered towards the end of the trajectory were found to be enriched in IPF lungs. Therefore, scAAnet was not only able to recover the trajectory-based archetype that was both IPF and myofibroblast enriched, but also identify one more archetype in fibroblasts, suggesting the potential of scAAnet on trajectory-based analysis at a higher resolution.’*

*Additionally, we have used data simulated under linear and bifurcation trajectories and showed scAAnet could identify GEPs corresponding to milestones alongside the simulated trajectories. We have added the following results and sentences about the potential of scAAnet on developmental data in S21 Fig, Results, S5 Text, and Discussion.*

*‘S21 Fig. scAAnet identified GEPs corresponding to milestones in simulated trajectory-based data. (a) Linear trajectory with three milestones colored by inferred GEP usage. (b) Bifurcation trajectory with four milestones colored by inferred GEP usage. Milestones are marked as black dots and backbone of the trajectory is shown in black curves.’*

*Results: ‘We explored the potential of scAAnet … and on developmental data simulated under linear and bifurcating trajectories in S5 Text.’*

*S5 Text: ’Another potential usage of scAAnet is for trajectory and pseudo-temporal analysis. Based on the definition of archetypal analysis, we argue that scAAnet can be used to fit developmental data following either a linear trajectory or a bifurcating trajectory. We used two simulated datasets from a benchmarking paper for trajectory inference methods, one with a linear trajectory and the other with a bifurcating trajectory, to showcase the usage of scAAnet on developmental data. We selected the number of archetypes based on the number of milestones in the simulated trajectory data (3 for linear and 4 for bifurcation). As shown in S21 Fig, for both datasets, scAAnet was able to identify GEPs that had good correspondence with known milestones along the trajectories. The closer the cells to a milestone, the more the contribution of the corresponding GEP to the expression profile of the cell. The results indicate the potential of scAAnet for analyzing developmental data with simple trajectory shapes.’*

*Discussion: ‘Fourth, although we have shown the potential of scAAnet on trajectory analysis when the topology is simple (e.g. linear or bifurcation), scAAnet cannot replace state-of-the-art trajectory inference methods because most developmental data contain a more complex topology (e.g. multifurcation or circular) [10]. Encompassing such data within a simplex would result in loss of resolution. Therefore, we recommend using scAAnet as a complement for trajectory analysis when the topology is simple, or data are measured within a relatively short developmental period.’*

- Related to the question above, one could imagine increasing K or using a larger convex hull would accommodate for more general shapes. In this sense, is there some trade-off involved in the use of archetypal constraint loss? Usually when different losses are combined, they are weighed differently with tuning parameters. Does the relative weight of reconstruction loss vs. archetypal loss affect any part of the results?

*Response: Thank you for your questions. The answer to your first question is yes, because we can ensure the archetypal structure and make inferred archetypes tighter to the data by adding a larger weight to the archetypal constraint loss. We have added a weight to the archetypal constraint loss and changed it to see how some metrics would change to answer your second question. Results are shown in S17 Fig and we have added the following paragraphs to Results and S2 Text separately.*

*‘S17 Fig. Influence of relative weight of the archetypal loss on scAAnet. (a) Change of the archetypal loss. (b) Change of the reconstruction loss. (c) Change of the total loss. (d) Performance on cell usage inference across different weights. (e) Performance on GEP inference across different weights. (f) Performance on reconstruction accuracy across different weights.’*

*Results: ‘The total loss of scAAnet is composed of both the archetypal loss and the reconstruction loss and one can change the relative weight of the two losses. Through simulation studies we found that the performance of scAAnet was robust to the change of the weight around 1, so we set the relative weight of the archetypal loss as 1 by default (see S2 Text for more discussions).’*

*S2 Text: ‘To study how the performance of scAAnet would be affected if we changed the relative weight of the archetypal loss, we used 13 different values (0.01, 0.02, 0.05, 0.1, 0.2, 0.5, 1, 2, 5, 10, 20, 50, and 100) as the weight on the archetypal loss and ran scAAnet 13 times with each value on a simulated dataset. From S17 Fig, we can see that the archetypal loss decreased as the weight increased, but the rate of change slowed down as the weight approached 1. In addition, the reconstruction loss and the total loss (reconstruction loss + weight * archetypal loss) did not change much because they were much larger than the archetypal loss in magnitude. For the three metrics assessing the performance of scAAnet in the second row, we can see that the accuracy of cell usage inference was robust to weights around 1, and the performance of scAAnet on GEP inference and reconstruction error were robust to all the chosen values of weight. Therefore, we think 1 is a good choice for weight and set it as scAAnet’s default value.’*

- It is stated that in simulation 50% of the samples were generated from the boundary of the simplex, which seems a bit contrived. What is the purpose of this construction? Would it be more realistic to include some outliers outside this simplex?

*Response: Thank you for your questions. ‘The purpose of this construction is to allow the existence of cells that don’t contain all the GEPs and show scAAnet is able to handle such cells.’ (We have added this sentence to Methods.) To support this, we have quantified the sparsity of inferred usage in the real data. Results are shown in S25 Fig and discussed in S8 Text.*

*‘S25 Fig. The level of sparsity of inferred usage in the real data. Barplots showing the distribution of the smallest inferred usage across cells grouped by annotated clusters in the pancreas islet dataset (a), the lung fibroblasts and myofibroblasts (b), and the microglial cells (c).’*

*‘We quantified the sparsity level of inferred usage in the three real datasets in this section. The smallest usage value for each cell was first obtained. Then the values across cells were binned into five windows ([0, 0.01), [0.01, 0.02), [0.02, 0.05), [0.05, 0.1), and [0.1, 1.0)). As shown in barplots in S25 Fig, we can observe that there existed a non-negligible percentage of cells in each dataset whose smallest usage was below 2% or even below 1%. The percentage was the largest for the pancreas islet dataset where many cell types were categorically delimited. Even for the lung dataset, the percentage of cells with smallest usage below 2% was over 10%. The results suggest the phenomenon that many cells contained only a subset of all the GEPs, so it is reasonable for us to simulate part of the data from the boundary of the simplex.’*

*For the second question, we want to first clarify how data were simulated. Data were simulated from NB distributions through Gamma-Poisson hierarchical models. Specifically, we first generated an archetype matrix and a usage matrix from Dirichlet distributions so that elements in each usage vector summed up to 1. Second, we obtained a mean expression matrix (cell*gene) from Gamma distributions, and finally generated an observed raw count matrix from Poisson distributions using the mean matrix as the parameter entry-wise. The mean expression matrix generated at the second step should exactly satisfy the condition that the mean vector for a cell was located within the simplex formed by the archetypes. However, since the observed raw count matrix is randomly generated, there could be cells whose observed expression vector could be located outside the simplex. Therefore, ‘outliers’ naturally existed at the raw count level.*

**Have the authors made all data and (if applicable) computational code underlying the findings in their manuscript fully available?**
The [PLOS Data policy](https://nam12.safelinks.protection.outlook.com/?url=https%3A%2F%2Fjournals.plos.org%2Fploscompbiol%2Fs%2Fmaterials-and-software-sharing&data=04%7C01%7Chongyu.zhao%40yale.edu%7Cc1b9a6d75d974a733a1008d9ba5117ab%7Cdd8cbebb21394df8b4114e3e87abeb5c%7C0%7C0%7C637745679898413080%7CUnknown%7CTWFpbGZsb3d8eyJWIjoiMC4wLjAwMDAiLCJQIjoiV2luMzIiLCJBTiI6Ik1haWwiLCJXVCI6Mn0%3D%7C3000&sdata=9JnNZyJfnwl6wC46OqTuHQ%2B8swXlCAq1QyVFBeS5CD0%3D&reserved=0) requires authors to make all data and code underlying the findings described in their manuscript fully available without restriction, with rare exception (please refer to the Data Availability Statement in the manuscript PDF file). The data and code should be provided as part of the manuscript or its supporting information, or deposited to a public repository. For example, in addition to summary statistics, the data points behind means, medians and variance measures should be available. If there are restrictions on publicly sharing data or code —e.g. participant privacy or use of data from a third party—those must be specified.

Reviewer #1: **No:** The method is on GitHub but "Analysis codes for generating results in this paper are available upon request by email." It would be good if these are published as well.

*Thank you for your feedback. We have uploaded analysis code on to the same GitHub page.*

Reviewer #2: Yes

Reviewer #3: Yes

References

1.	Lopez R, Regier J, Cole MB, Jordan MI, Yosef N. Deep generative modeling for single-cell transcriptomics. Nat Methods. 2018;15(12):1053-8.

2.	Eraslan G, Simon LM, Mircea M, Mueller NS, Theis FJ. Single-cell RNA-seq denoising using a deep count autoencoder. Nat Commun. 2019;10(1):390.

3.	Keller SM, Samarin M, Wieser M, Roth V, editors. Deep archetypal analysis. German Conference on Pattern Recognition; 2019: Springer.

4.	Li WV, Li JJ. An accurate and robust imputation method scImpute for single-cell RNA-seq data. Nature communications. 2018;9(1):1-9.

5.	Pierson E, Yau C. ZIFA: Dimensionality reduction for zero-inflated single-cell gene expression analysis. Genome biology. 2015;16(1):1-10.

6.	Blei DM, Laﬀerty JD. Topic models. Text mining: Chapman and Hall/CRC; 2009. p. 101-24.

7.	Blei DM. Probabilistic topic models. Communications of the ACM. 2012;55(4):77-84.

8.	Hofmann T, editor Probabilistic latent semantic indexing. Proceedings of the 22nd annual international ACM SIGIR conference on Research and development in information retrieval; 1999.

9.	Blei DM, Ng AY, Jordan MI. Latent dirichlet allocation. the Journal of machine Learning research. 2003;3:993-1022.

10.	Saelens W, Cannoodt R, Todorov H, Saeys Y. A comparison of single-cell trajectory inference methods. Nature biotechnology. 2019;37(5):547-54.

11.	Satija R, Farrell JA, Gennert D, Schier AF, Regev A. Spatial reconstruction of single-cell gene expression data. Nature biotechnology. 2015;33(5):495-502.
