# Supplementary material for: Non-linear archetypal analysis of single-cell RNA-seq data by deep autoencoders
Source: PLoS Comput Biol. 2022 Apr 1;18(4):e1010025. doi: 10.1371/journal.pcbi.1010025 (PMC9007392; doi:10.1371/journal.pcbi.1010025)
Supplement: Attachment — Submitted filename: Response to reviewers.docx [file pcbi.1010025.s058.docx]

**Authors’ Responses**

**Point-by-point responses to the reviewers’ comments:**

Reviewer #1:

The authors have addressed most of my comments. I would like to see a few supplementary figures added to improve the interpretation of the GEPs.

Specifically, regarding the inferred GEP matrix. The GEPs corresponds to the archetypes, which, as the authors describe, represent “pure types or extrema of the real data”.

In the pancreatic data set, most cell types are primarily associated with one GEP, to the extent that we might even annotate that GEP using the cell type name. Epsilon cells are associated with GEP 3 (gamma) and GEP 5(delta), and the authors provide the interpretation that Epsilon cells are similar to both gamma and delta cells.
How does one interpret that the beta cells having very high usage of either GEP2 or GEP7? The authors compared GEP2 and GEP7 and concluded that these contain different top genes, and the genes are associated with different GO terms. Does this suggest that there are two different subtypes of beta cells, one subtype resembling archetype 2 and another resembling archetype7?

*Response: Thank you for your questions. Yes, you are right that our results on the pancreatic dataset suggested there should exist two ‘subtypes’ of beta cells. Instead of saying there were two ‘subtypes’ of beta cells, we would prefer to say there were two substates of beta cells. Because as shown in S24 Fig a, most of the beta cells had the largest inferred cell usage below 0.6, indicating that many beta cells used both GEP 2 and GEP 7 and many of them were not a ‘pure’ GEP 2 or GEP 7-using cell. By conducting GO enrichment analysis on the top 20 DEGs of each archetype, we found GEP 2 was responsible for the traditional functions of beta cells, such as peptide hormone secretion and insulin secretion, and GEP 7 was functionally related to the stress response. These findings were consistent with the observations in the original paper [1]. However, in the original paper, the authors detected these two ‘subtypes’ through a more exploratory type of analyses. More specifically, they performed PCA on beta cells and found genes positively correlated with the value of PC1 were functional beta genes and genes negatively correlated with the value of PC1 were involved in the stress response. We think the benefit of our method is that it provides a more systematic and principled way of identifying the heterogeneity within a cell type.*

*We have added the following sentences to S7 Text and our main text based on the above response.*

*S7 Text: “Note that for beta cells in the pancreatic islet dataset, since we detected two GEPs there (GEP 2 and GEP 7), the barplot indicates that many beta cells used both GEP 2 and GEP 7 and many of them were not a ‘pure’ GEP 2 or GEP 7-using cell.”*

*Main Text Discussion: “However, in the original paper, the authors detected these two ‘subtypes’ through a more exploratory type of analyses. More specifically, they performed PCA on beta cells and found genes positively correlated with the value of PC1 were functional beta genes and genes negatively correlated with the value of PC1 were involved in the stress response. We think the benefit of our method is that it provides a more systematic and principled way of identifying the heterogeneity within a cell type.”*

The authors provide in Fig 10 the top 20 DEGs for GEP2 and GEP 7. But lacking an overlap between the top 20 genes does not directly reflect how much GEP2 and GEP7 differ. Can the authors provide a heatmap for the GEP matrix (as described on Line 178-179)? The usage of the GEPs are visualized (for example, in Fig 5c). There are 2000 HVGs from 1937 cells, so the GEP matrix is not much larger than the GEP usage matrix. A heatmap can provide a rather straightforward view of the GEPs, and help the reader interpret the GEPs, in addition to the top DEG and GO analysis that the authors already provide.

It would be useful to add these in supplementary figures:
• For the simulated data, heatmaps of the true GEP matrix and the inferred GEP matrix(after re-arranging the GEPs to best match the true order)
• For the real data sets, heatmaps of the inferred GEP matrix

*Response: Thank you for your suggestions. For the pancreatic dataset, in the previous version of our manuscript, we showed a heatmap of normalized gene scores for known marker genes across 10 GEPs in Fig 5e. The reason why we didn’t show the entire heatmap of all genes was because each GEP vector summed up to one (the vector length was 2,000 in contrast to a vector length of just 10 for each usage vector in the pancreatic dataset) and many of them were dominated by a limited number of genes (see the ranked gene score plot below). As a result, if we plotted all the genes (see the entire normalized gene score heatmap below), we can observe that most genes had a close-to-zero score and the visualization would not be very informative. Instead, we can take the union of all top 20 DEGs across all the GEPs and draw similar heatmaps for different datasets.*

*Ranked gene score plot (for review purpose only)*

*Normalized gene scores heatmap of 2,000 HVGs (for review purpose only)*

*Below are normalized gene scores for a simulated dataset and the three real datasets, and we put them together as S26 Fig. Note that there were no overlaps among the top 20 DEGs from different GEPs in all the datasets.*

*S26 Fig. Heatmaps of normalized gene scores of the top 20 DEGs across different GEPs (a) in a simulated dataset, where top is from the true GEP matrix and bottom is from the inferred the GEP matrix (rearranged GEPs to match the order of true GEPs by Pearson correlation); (b) in the pancreatic islet dataset (where the bottom if a subset of the top figure without IAPP for a better resolution); (c) in the lung dataset; (d) in the prefrontal cortex dataset.*

Minor:
The DEG test for archetypes
Line 799 : is $m_i$ the library size? To be consistent with the notation used earlier, maybe it’s better to use $l_i$ again.

*Response: Thank you for your suggestion. Yes, $m_i$ is the library size. We have changed the notation to $l_i$ and added the following sentence:*

*‘*where $x_{ig}$ is the raw count of gene *g* in cell *i*, $U_{ik}$ is the inferred cell usage of GEP *k* in cell *i* and $l_{i}$ is the library size of cell *i*.*’*

In the results for the lung fibroblast and myofibroblast cells: “GEP 3 is the one not only cell-type-specific but also disease-related.” However, Fig 7 seems to suggest that in Myofibroblasts, both GEP2 and GEP3 usage are disease related?

*Response: Thank you for your question. Here, we regarded a GEP as disease-specific or cell-type-specific when the usage was significantly high in a disease compared to control or high in a cell type compared to others. As we can see in Fig 7e and 7f, GEP 2 was a myofibroblast-specific GEP, however, the overall usage level of GEP 2 in this cell type was high in both control and COPD group but was low in the IPF group. This was due to the nature of compositional data. Since the usage of GEP 3 was high in myofibroblasts and in IPF patients, it’s expected that the usage level of GEP 2 was low in the same group of cells as all the usages need to sum to 1. Therefore, we didn’t regard it as a IPF or COPD specific archetype.*

*Reference*

*[1] Baron M, Veres A, Wolock SL, Faust AL, Gaujoux R, Vetere A, et al. A single-cell transcriptomic map of the human and mouse pancreas reveals inter-and intra-cell population structure. Cell systems. 2016;3(4):346-60. e4.*
